# Investigation of the Pyridinium Ylide—Alkyne Cycloaddition as a Fluorogenic Coupling Reaction

**DOI:** 10.3390/molecules21030332

**Published:** 2016-03-10

**Authors:** Simon Bonte, Ioana Otilia Ghinea, Rodica Dinica, Isabelle Baussanne, Martine Demeunynck

**Affiliations:** 1 Department of Pharmacochemistry, Université Grenoble Alpes, CNRS, DPM UMR 5063, F-38041 Grenoble, France; simontlse.12@gmail.com; 2 Department of Chemistry, Physics and Environment, Faculty of Science and Environment, “Dunarea de Jos” University of Galati, 111 Domneasca Street, 800201 Galati, Romania; otilia.ghinea@gmail.com (I.O.G.); rodicad@yahoo.com (R.D.)

**Keywords:** indolizine, coupling reaction, ylide, dipolar cycloaddition

## Abstract

The cycloaddition of pyridinium ylides with alkynes was investigated under mild conditions. A series of 13 pyridinium salts was prepared by alkylation of 4-substituted pyridines. Their reactivity with propiolic ester or amide in various reaction conditions (different temperatures, solvents, added bases) was studied, and 11 indolizines, with three points of structural variation, were, thus, isolated and characterized. The highest yields were obtained when electron-withdrawing groups were present on both the pyridinium ylide, generated *in situ* from the corresponding pyridinium salt, and the alkyne (X, Z = ester, amide, CN, carbonyl, *etc.*). Electron-withdrawing substituents, lowering the acid dissociation constant (pKa) of the pyridinium salts, allow the cycloaddition to proceed at pH 7.5 in aqueous buffers at room temperature.

## 1. Introduction

**Scheme 1:**
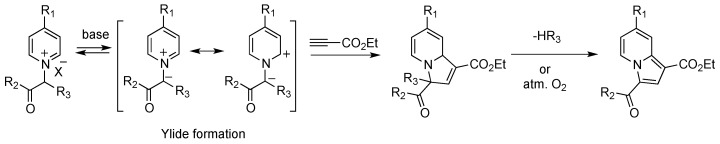
General mechanism.

We have been interested in the indolizine chemistry for several years. Indolizine is a nitrogen-containing bicyclic heterocycle, and its derivatives display interesting biological [1,2,3,4,5] and optical properties [6,7,8,9,10,11,12,13] (fluorescence and circular dichroism [14,15]). Indolizines have been used as biomarkers [16] and in the fluorescent labeling of carbon nanotubes [17] and graphene [18], for instance. Among the main routes of preparation that have been designed, we can cite the metal-catalyzed cyclization of 2-alkynylpyridines [19,20,21,22,23,24,25] or 2-pyridine alkynyl carbinols [26,27,28,29,30,31]. The most common metal-free methodology [2,32,33] involves the 1,3-dipolar cycloaddition of pyridinium ylides with alkynes (Scheme 1) [2,34,35,36,37,38,39]. The dihydroindolizines thus formed spontaneously, aromatize under air oxidation. This chemistry has been the subject of a large number of publications, focusing, in particular, on the formation and reactivity of the ylides [34,35,40,41,42,43,44] and on the mechanism of the cycloaddition [33]. More recently, improvements in the reaction conditions have been reported, by using oxidant-free cycloaddition to alkenes [32], one-pot [38], microwave-activated [34,35], or biocatalyzed processes [36]. Note that most of the work reported so far in the literature involved *N*-benzoylmethylpyridinium-derived ylides [45,46]. The purpose of the present work was to explore the potency of the pyridinium ylide-alkyne cycloaddition as a click–type coupling reaction.

The key points to investigate were the effectiveness of this cycloaddition in mild conditions (room temperature, neutral or near neutral conditions), ease of accessibility of the reactants, and their practical pre- or post-functionalization. For all these reasons, we decided to prepare a series of ylide precursors (pyridinium salts) and to compare their reactivity in the presence of alkynes in various conditions. We first aimed to select the best partners for the application as coupling methodology. To achieve this goal, we modified the structure of the ylide precursors: reactive or easily modified substituents were introduced on the pyridinium salts (R_1_ group in Scheme 1) and ester or amide groups were used to stabilize the ylide (R_2_ = RO- or RNH-, in Scheme 1) in place of the benzoyl group usually reported in the literature. The structure optimization of the reactants allowed the cycloaddition to proceed at room temperature in very mild conditions including pH 7.5 aqueous buffers, yielding indolizines with three possible points of functionalization. 

## 2. Results

### 2.1. Preparation and Characterization of the Pyridinium Salt

We first prepared a series of 13 pyridinium salts. To keep the symmetry of the starting molecule and to prevent formation of regioisomers after cycloaddition, substituents were only introduced at position 4 of the pyridine ring [37].

The pyridinium salts were obtained by alkylation of the pyridine derivatives with methyl 2-bromoacetate (compounds **1**–**7**), 2-iodoacetophenone derivatives (compounds **8** and **9**), or 2-bromo-*N*-propylacetamide (compound **10**) in acetone under ultrasound activation. In the case of alkylation with diethyl 2-iodomalonate to prepare the pyridinium salts containing two carboxylic esters (compounds **11**–**13**, R_3_ = CO_2_Et), very low yields were observed under these conditions. The reaction was improved by using the diethyl 2-iodomalonate in large excess. The structures, yields, pKa and ^1^H-NMR data are collected in Table 1.

As previously observed [47,48], the electronic nature of the substituent present at position 4 modulates the pKa values, and the pKa variation correlates well with the Hammett constant of the R_1_ group. The electron-withdrawing groups decrease the pKa values (compare **4**–**7** *versus* **1**). This effect was more pronounced with mesomeric (CN or COCH_3_) than inductive (CF_3_) withdrawing substituents. The effect of the nature of R_1_ has been studied [49] and seemed more prominent for the phenacyl analogues [47], however the measurements were made in different conditions (ylides dissolved in methanol). In another study, the deprotonation of pyridinium salts was studied by NMR in DMSO in the presence of a strong base [48]. The authors found that the effect on the deprotonation of the ring substituents was greater than the effect of the methylene substituent. 

The proton NMR spectra of 4-substituted pyridinium salts are characterized downfield by two multiplets for H-2/H-6 and H-3/H-5, the latter being more shielded, and a singlet for the CH_2_ generally found between 4.4 and 6.6 ppm. The correlation between the presence of the electron-withdrawing group R_1_ and pKa (deprotonation and ylide formation) is reflected by higher δ value for the CH_2_ signal. The integration of this singlet was lower than expected with compounds **4**–**6** and this signal may also be lacking (see **11** and **12**) probably due to high H/D exchange rate in CD_3_OD. We observed in the spectrum of the 4-acetylpyridinium salt **6** in CD_3_OD, the presence of a second set of shielded signals, not found in DMSO-*d*_6_, which was attributed to the formation of hemiketal or ketal derivatives in this solvent and that increased with time spent in solution (the simulated spectra of the different species were in agreement with the experimental data).

### 2.2. Reactivity Studies

The reactivity of the different salts with alkynes was compared using a reference reaction: methanol was chosen as solvent, ethyl propiolate as dipolarophile, and K_2_CO_3_ for ylide generation. The reaction mixtures were stirred at 25 °C for 18 h, and the indolizines were isolated. The ease of isolation and purification is an important point for the usefulness of the reaction. Therefore, in the following tables, we give and discuss the yields of isolated indolizines.

As known in the literature, the cycloaddition is fully regioselective, the ethyl and methyl esters being found in positions 1 and 3, respectively, in our study and easily identified.

#### 2.2.1. Influence of the R_1_ Substituent

We first studied the importance of the nature of R_1_ on the reactivity (Table 2). Introduction of electron-withdrawing groups on the pyridine ring clearly favored the cycloaddition. Higher yields (77% and 81%) were obtained in the presence of electron-withdrawing mesomeric groups (COCH_3_ or CN, respectively) that efficiently stabilize the negative charge of the ylides by delocalization. In the case of the CF_3_ substituent (entry 6), the NMR data showed the presence of the desired indolizine **19** and of the corresponding dimethyl carboxylate, formed by trans-esterification of the ethyl ester at position 1 by methanol. To selectively prepare **19**, the reaction should be performed in ethanol or in a non-nucleophilic solvent such as DMF (cf. part 2.2.2). Under these mild conditions, the 4-amino or amido-substituted pyridinium salts (entries 2 and 3), which display the lowest Hammett constant, did not react.

From these experiments, compounds **6** and **7** were selected for further studies.

#### 2.2.2. Influence of the Solvent and of the Nature of the Added Base

To evaluate the importance of the solvent on the cycloaddition efficiency, the reactions were performed in parallel in DMF, a polar non-nucleophilic solvent, and methanol. Three bases, NH_4_OH, NEt_3_, and K_2_CO_3_ were also tested. The data are collected in Table 3.

K_2_CO_3_ appeared as the most efficient base in both solvents (entries 1 and 5), and DMF emerged as the best choice to perform reactions with **6** (R_1_ = COCH_3_). The reaction proceeded quickly in this solvent with good yield obtained in 1 h (entry 5). Increasing the reaction time to 5 h (entry 8) did not significantly improve the yield. This solvent effect was also observed with the 4-cyano analog **7**, for which both solvents may be alternatively used. Still, the reaction remained faster in DMF than in methanol (compare entries 11 and 13). The reaction was attempted at a higher temperature in methanol (entries 12 and 14), but leading to the formation of side-products including those resulting from trans-esterification.

The reaction was also performed in pH 7.5 phosphate buffer (entry 15). The pyridinium salt **7** was soluble in aqueous solution, but the hydrophobic nature of the ethyl propiolate was a severe limitation. Nevertheless, we were thrilled to isolate the resulting indolizine **20** in 24% yield. This result indicated that the pKa of the salt (8.16 for **7**) was compatible with the partial formation of the ylide under these conditions, thus allowing the cycloaddition reaction. It should be noted that, to our knowledge, dipolar cycloaddition involving a pyridinium ylide in neutral aqueous solution has not been reported so far. 

In an effort to increase the reactivity in water, the reaction was performed with compounds **11**–**13** that displayed lower pKa values and would mainly exist as ylides at pH 7.5. The reactions were performed in pH 7.5 buffer solutions. For solubility reasons, the first attempts were made at 40 °C in Tris buffer. As indicated in Table 4, the cyclization was highly dependent on the nature of the substituent at position 4. There was no reaction with the unsubstituted pyridinium **11** (entry 1), and a low yield in indolizine **22** was obtained from the 4-acetyl pyridinium **12** (entry 2). The 4-cyanopyridinium **13** yielded the indolizine **23** in a reasonable 40% yield. As we had previously observed a negative effect of temperature on yields, the reaction was then performed at 25 °C under vigorous stirring and, as a result, the yield jumped to 63% (entry 4). Replacing Tris buffer with phosphate buffer (entry 5) had a negative effect on the yield that dropped to 42%. For comparison, the reactivity of **13** in organic solvents was investigated (entries 6 and 7). A very strong solvent effect was observed. While no formation of **23** occurred in methanol, it was isolated in excellent yield in DMF. The difference in reactivity between **12** and **13** in Tris buffer may be due to the unfavorable formation of the hydrate form of **12**. However, these first data confirmed the feasibility of this dipolar cycloaddition in aqueous solutions starting with 4-cyanopyridinium salts **7** and **13**.

#### 2.2.3. Effect of the R_2_ Group 

To extend the scope of the reaction, it was also important to compare the reactivity of pyridinium salts containing various methylene R_2_ substituents. As shown in Table 5, the yield in 7-cyanoindolizine was higher starting from the methyl ester **7** than from benzoyl derivatives **8** or **9**. The presence of the nitro group on the phenyl ring, lowering the pH of ylide formation, slightly increased the yield (compare entries 2 and 3).

An interesting result was obtained with the amide containing pyridinium salt **10**. The yield of indolizine **26** was moderate (62%), but this result was important as it gives an interesting alternative to the ester as a potential reactant, and this approach has not been reported so far in the literature. 

#### 2.2.4. Influence of the Dipolarophile

The last parameter to evaluate was the nature of the dipolarophile. As shown in Table 6, the reaction worked also well with propiolic amide such as **27**, giving the corresponding indolizine **28** in reasonable (not optimized) yield. With substituted propiolate, such as **29**, a complex mixture of indolizines was obtained as shown by the presence of several fluorescent spots on TLC. No reaction was observed with less activated alkynes, as exemplified by **30** or **31**. 

Again, it is important to emphasize the mild conditions used in this study. Simple pyridinium ylides were shown to react at high temperatures with isolated alkynes [39]. However the lack of reactivity with isolated alkynes at room temperature may be useful as it allows the introduction of an isolated triple bond in the reactants for further orthogonal reactions. 

## 3. Discussion

To summarize, the highest yields of indolizines were obtained when both partners of the reaction, the pyridinium salt (or the corresponding ylide) and the triple bond, were substituted with a strong electron-withdrawing group (acetyl, cyano, ester, or amide). The mechanism of the cycloaddition has been discussed in the literature. The reactions of pyridinium ylides with propiolates are generally described as concerted OM-controlled reactions [33,40,43,44]. Matsumoto [41] also reported the inverse electron-demand cycloaddition of cyclooctyne with pyridinium bis(methoxycarbonyl)methylides similar to **11**. However, Shang and colleagues [39] found that, in the reaction with simple alkynes in DMF–K_2_CO_3_ at high temperature (120 °C), the presence of electron-donating or –withdrawing substituents on the ylides significantly lowered the yields in indolizines. 

**Scheme 2:**
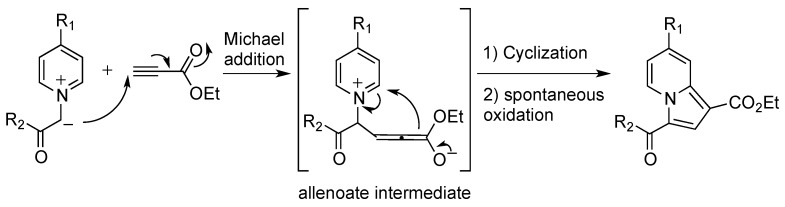
Alternative two-step mechanism.

The importance of electron-withdrawing groups on both reactants is in favor of the two-step mechanism depicted in Scheme 2, involving the Michael addition of the ylide to the triple bond, with formation of zwitterionic allenoate intermediate followed by the intramolecular 5-endo trig cyclization. However, one also has to keep in mind that the very first step is not the cycloaddition itself, but the formation and stabilization of the reactive ylides that are also favored by electron-withdrawing groups.

In conclusion, the reaction of pyridinium ylides with propiolic acid derivatives was explored in the perspective of its use as coupling reaction. The reaction was investigated by varying a set of parameters, *i.e.*, the nature of the substituents, the reaction conditions, and the nature of the dipolarophile. First of all, the regioselectivity of the reaction is a positive aspect for this application. The presence of an electron-withdrawing group at position 4 of the pyridinium salt (exemplified with 4-cyano or 4-acetyl derivatives) allowed the reaction with propiolic ester or amide to proceed in mild conditions in a variety of solvents. The reaction progression was easily monitored by TLC, due to the fluorescence of the newly-formed indolizine. This point is of major interest is that, so far, most pro-fluorescent click reactions involve the use of added fluorogenic or fluorescent heterocycles [50,51].

A second key result was the reactivity in neutral aqueous solutions at room temperature. Indeed, the presence of the electron-withdrawing group at position 4 played an essential role in the formation and stabilization of the reactive ylides in these conditions. The main limitation appeared to be the water solubility of the propiolic ester.

This study allowed us to select the best partners and conditions for a highly modular pro-fluorescent click-type coupling reaction. The reactants include a pyridine containing an electron-withdrawing group (CN, COR, CO_2_R, CONHR, *etc.*) at position 4, a 2-bromo-acetyl ester or amide, and a propiolic ester or amide. Interestingly, this methodology appears complementary to the tetrazine-alkene reaction [52,53] involving an electron-rich dipolarophile, but yielding non-fluorescent compounds.

## 4. Experimental Section 

### 4.1. Material and Methods

Melting points were determined using a Reichert Thermovar apparatus (Depew, NW, USA) and are uncorrected. NMR spectra were recorded on the Bruker Avance 400 spectrometer (Bruker Corporation, Billerica, MA, USA) of the “Fédération de Recherche” ICMG (FR2607) platform, using the solvent as the internal reference; the chemical shifts are reported in parts per million (ppm) units. High-resolution mass spectra (HRMS) were performed on a Bruker maXis mass spectrometer Q-TOF (Bruker Corporation) by the “Fédération de Recherche” ICOA/CBM (FR2708) platform. Reversed-phase HPLC was performed with a μ-bondapak-C18 analytical column (Waters Corporation, Milford, MA, USA). A Waters chromatographic system was used, with two M-510 pumps and a photodiode array detector Waters 996 using Millenium 32 software. A linear gradient from 0 to 100% methanol in H_2_O pH 2.5 (phosphoric acid), 2 mL/min flow rate, was used.

The reagents were purchased from Sigma Aldrich and were used without further purification. *N*-Benzylprop-2-ynamide **27** was prepared by biocatalyzed reaction between benzylamine and ethyl propiolate as reported recently by us [54]. Ethyl 3-ethynylbenzoate **30** was prepared by esterification of 3-ethynylbenzoic acid following reported procedure [55]. 

Calculator Plugins were used for structure property prediction and calculation, Marvin 6.0.2 , 2013, ChemAxon (http://www.chemaxon.com).

Copies of the NMR spectra and HPLC chromatograms of the new compounds may be found in the Supplementary Materials.

### 4.2. General Methods for the Synthesis of N-Heterocyclic Salts

**Method A**: The pyridine derivative (1 eq.) and the alkylating reagent (1.5 eq) were dissolved in dry acetone (2 mL for 1 mmol of pyridine derivative). The reaction mixture was stirred in an ultra-sound bath for 5 h to 10 h, depending on the nature of substituent present on the pyridine. The temperature of the bath was kept under 50 °C by adding ice is necessary. Then, a non-polar solvent (3 to 5 mL of Et_2_O or DCM) was added, and the quaternary salt that deposited was filtered off, and washed with DCM. 

**Method B**: The reactions between the pyridine derivatives and diethyl iodomalonate were performed in acetone in the presence of a large excess of reactant, the mixture being stirred at room temperature for two days. Then, Et_2_O was added to the flask and the quaternary hygroscopic salt was filtered off and washed with DCM and/or Et_2_O.

*1-(2-Methoxy-2-oxoethyl)pyridinium bromide* (**1**) was prepared according to the general method A from pyridine (1 mmol) and methyl 2-bromoacetate. The resulting pyridinium **1** was obtained in 71% yield (165 mg) as a white powder. mp 167–168 °C [lit. [56] 174–175 °C]; ^1^H-NMR (400 MHz, CD_3_OD) δ 9.01 (dd, 2H, *J* = 6.8, 1.2 Hz), 8.74 (m, 1H), 8.23 (t, 2H, *J* = 7.6, 6.8 Hz), 5.66 (s, 2H), 3.90 (s, 3H). 

*4-Amino-1-(2-methoxy-2-oxoethyl)pyridinium bromide* (**2**) was prepared according to the general method A from 4-aminopyridine (1 mmol) and methyl 2-bromoacetate. The resulting pyridinium salt **2** was obtained in 83% yield (205 mg) as a white powder. mp 268–269 °C [lit. [57] 175–176 °C]; ^1^H-NMR (400 MHz, CD_3_OD) δ 8.09 (dd, *J* = 6 Hz, 2H), 6.92 (dd, *J* = 6 Hz, 2H), 5.12 (s, 2H), 3.86 (s, 3H). 

*4-Acetamido-1-(2-methoxy-2-oxoethyl)pyridinium bromide* (**3**) was prepared according to the general method A from 4-acetamidopyridine (1 mmol) and methyl 2-bromoacetate The resulting pyridinium salt **3** was obtained in 81% yield (234 mg) as a white powder. mp 104–106 °C; ^1^H-NMR (400 MHz, DMSO-*d*_6_) δ 11.61 (s, 1H), 8.74 (d, 2H, *J* = 7.6 Hz), 8.14 (d, 2H, *J* = 7.2 Hz), 5.51 (s, 2H), 3.78 (s, 3H), 2.26 (s, 3H); ^13^C-NMR (100 MHz, DMSO-*d*_6_) δ 171.1, 167.3, 152.5, 146.6, 114.3, 58.6, 53.0, 24.7; HRMS (ESI) *m/z* calcd for C_10_H_13_N_2_O_3_ 209.0926, obsd 209.0919.

*4-Trifluoromethyl-1-(2-methoxy-2-oxoethyl)pyridinium bromide* (**4**) was prepared according to the general method A from commercial 4-trifluoromethylpyridine (2 mmol) and methyl 2-bromoacetate The resulting quaternary salt **4** was obtained in 54% yield (162 mg) as a yellow powder. mp 127–130 °C; ^1^H-NMR (400 MHz, CD_3_OD) δ 9.39 (d, 2H, *J* = 6.8 Hz), 8.66 (d, 2H, *J* = 6.8 Hz), 5.83 (s, 2H), 3.94 (s, 3H); ^13^C-NMR (100 MHz, CD_3_OD) δ 165.9, 148.6 (2C), 145.9, 145.5, 124.6 (2C), 122.5, 119.8, 61.0, 52.8; HRMS (ESI) *m/z* calcd for C_9_H_9_F_3_NO_2_ 220.0577, obsd 220.0580.

*4-(N-Propylcarbamoyl)-1-(2-methoxy-2-oxoethyl)pyridinium bromide* (**5**) was pepared according to the general method A from 4-*N*-propylcarbamoylpyridine (0.9 mmol) and methyl 2-bromoacetate The resulting quaternary salt **5** was obtained in 80% yield (231 mg) as an orange powder. mp 136–138 °C; ^1^H-NMR (400 MHz, CD_3_OD) δ 9.19 (dd, 2H, *J* = 6.4 Hz); 8.52 (dd, 2H, *J* = 6.4 Hz), 5.75 (s, 2H), 3.92 (s, 3H), 3.46 (t, 2H, *J* = 7.4 Hz), 1.71–1.76 (m, 2H), 1.05 (t, 3H, *J* = 7.4 Hz); ^13^C-NMR (100 MHz, CD_3_OD) δ 167.6, 164.0, 151.8, 148.6 (2C), 1267.0 (2C), 61.8, 54.1, 43.3, 23.4, 11.7; HRMS (ESI) *m/z*: [M + H]^+^ calcd for C_12_H_17_N_2_O_3_ 237.1234, obsd 237.1232.

*4-Acetyl-1-(2-methoxy-2-oxoethyl)pyridinium bromide* (**6**) was prepared according to the general method A from 4-acetylpyridine (4.1 mmol) and methyl 2-bromoacetate. The resulting quaternary salt **6** was obtained in 64% yield (716 mg) as a red powder. mp 149–150 °C; ^1^H-NMR (400 MHz, CD_3_OD) δ 9.35 (dd, 2H, *J* = 6.8 Hz); 8.63 (dd, 2H, *J* = 6.8 Hz); 5.88 (s, 2H, CH_2_); 3.80 (s, 3H, OCH_3_), 2.78 (s, 3H, CH_3_); ^13^C-NMR (100 MHz, DMSO-*d*_6_) δ 195.5, 166.6, 149.2, 147.8 (2C), 125.7 (2C), 60.4, 53.3, 27.5; HRMS (ESI) *m/z* calcd for C_10_H_12_NO_3_ 194.0812, obsd 194.0810.

*4-Cyano-1-(2-methoxy-2-oxoethyl)pyridinium bromide* (**7**) was prepared according to the general method A from 4-cyanopyridine (1 mmol) and methyl 2-bromoacetate. The resulting pyridinium salt **7** was obtained in 60% yield (154 mg) as a white powder. mp 208–209 °C [lit. [58] 181–183 °C]; ^1^H-NMR (400 MHz, CD_3_OD) δ 9.29 (dd, 2H, *J* = 5.4 Hz), 8.64 (dd, 2H, *J* = 5.4 Hz), 5.75 (s, 2H), 3.92 (s, 3H).

*4-Cyano-1-(2-oxo-2-phenylethyl)pyridinium iodide* (**8**) was prepared according to the general method B from commercial 4-cyanopyridine (2 mmol) and 2-iodo-acetophenone. The resulting pyridinium salt **8** was obtained in 70% yield (493 mg) as a red powder. mp 132–134 °C [lit. [47] 114–118 °C]; ^1^H-NMR (400 MHz, DMSO-*d*_6_) δ 9.30 (d, 2H, *J* = 6.9 Hz), 8.84 (d, 2H, *J* = 6.9 Hz), 8.09 (dd, 2H, *J* = 8.5, 7.1 Hz), 7.80–7.85 (m, 1H), 7.67–7.72 (m, 2H), 6.61 (s, 2H).

*4-Cyano-1-(2-oxo-2-(para-nitrophenyl)ethyl)pyridinium iodide* (**9**) was prepared according to the general method A from commercial 4-cyanopyridine (1 mmol) and 2-iodo-4-nitroacetophenone. The resulting quaternary salt **9** was obtained in 60% yield (239 mg) as a yellow powder. mp 182–184 °C [lit. [47] 187–189 °C]; ^13^C-NMR (100 MHz, DMSO-*d*_6_) δ 189.2, 150.7, 147.6 (2C), 138.0, 130.8 (2C), 129.8 (2C), 128.1, 124.2 (2C), 114.8, 67.3. 

*4-Cyano-1-[(N-propylcarbamoyl)methyl]pyridinium bromide* (**10**) was prepared according to the general method A from 4-cyanopyridine (1 mmol) and 2-bromo-*N*-propylacetamide. The resulting pyridinium-salt **10** was obtained in 60% yield (160 mg) as a yellow powder. mp 185–186 °C; ^1^H-NMR (400 MHz, CD_3_OD) δ 9.24 (d, 2H, *J* = 6.8 Hz), 8.59 (d, 2H, *J* = 6.8 Hz), 5.60 (s, 2H), 3.26 (t, 2H, *J* = 7.2 Hz), 1.58–1.64 (m, 2H), 0.99 (t, 3H, *J* = 7.2 Hz); ^13^C-NMR (100 MHz, CD_3_OD) δ 165.0, 149.1 (2C), 131.7 (2C), 130.4, 115.3, 63.9, 42.9, 23.5, 11.8; HRMS (ESI) m/z: [M + H]^+^ calcd for C_11_H_14_N_3_O 204.1131, obsd 204.1132.

*1-(1,3-Diethoxy-1,3-dioxopropan-2-yl)pyridinium iodide* (**11**) was prepared according to the general method B from pyridine (5.5 mmol) and ethyl 2-iodomalonate. The resulting quaternary salt **11** was obtained in 89% yield (1.80 g) as a white powder. mp 151–152 °C [lit. [59] 154–155 °C]; ^1^H-NMR (400 MHz, CD_3_OD) δ 9.20 (t, 2H, *J* = 7.2 Hz), 8.81–8.85 (m, 1H), 8.28 (dd, 2H, *J* = 7.2 Hz, 8 Hz), 4.37–4.52 (m, 4H), 1.40 (t, 6H, *J* = 7.2 Hz).

*4-Acetyl-1-(1,3-diethoxy-1,3-dioxopropan-2-yl)pyridinium iodide* (**12**) was prepared according to the general method B from 4-acetylpyridine (4 mmol) and ethyl 2-iodomalonate. The resulting pyridinium-salt **12** was obtained in 99% yield (1.66 g) as a yellow powder. mp 130–131 °C [lit. [37]: 196–198 °C]; ^1^H-NMR (400 MHz, CD_3_OD) δ 9.36 (d, 2H, *J* = 7.0 Hz), 8.61 (d, 2H, *J* = 7.0 Hz), 4.39–4.51 (m, 4H), 2.84 (s, 3H), 1.41 (t, 6H, *J* = 7.1 Hz).

*4-Cyano-1-(1,3-diethoxy-1,3-dioxopropan-2-yl)pyridinium iodide* (**13**) [37] was prepared according to the general method B from 4-cyanopyridine (4.8 mmol) and ethyl 2-iodomalonate. The resulting pyridinium-salt **13** was obtained in 40% yield (750 mg) as a white powder. mp 120–122 °C [lit. [37] 240 °C decomposed]; ^13^C-NMR (100 MHz, CD_3_OD) δ 163.3 (2C), 151.6, 149.7 (2C), 132.0, 131.4 (2C), 115.2, 65.8 (2C), 14.2 (2C).

### 4.3. General Methods for the Ylide-Alkyne Cycloaddition

Method C: The cycloaddition was performed with 1 eq. of the quaternary salt, 1.1 eq. of the alkyne derivative, and 1 eq. of K_2_CO_3_ in methanol or DMF. The pH of the solution was close to 9. The reaction mixture was stirred at room temperature under air atmosphere for 18 h. Then, water was added and the corresponding indolizine was precipitated, filtered off and washed with water.

**Method D**: The cycloaddition was performed in Tris-buffer pH 7.5 (Tris-buffered saline tablets from sigma, one tablet dissolved in 15 mL of deionized water produces 50 mM Tris-HCl, 150 mM sodium chloride) with 1 eq. of the quaternary salt and 1.1 eq of the alkyne derivative. The reaction mixture was stirred at 40 °C under air atmosphere for 18 h. Then, the reaction mixture cooled in an ice bath, and the precipitate that formed was filtered off and washed with water.

*1-Ethyl 3-methyl indolizine-1,3-dicarboxylate* (**14**). The reaction was performed with **1** (100 mg) and ethyl propiolate in methanol, following method C. The indolizine **14** was obtained in 59% yield (63 mg) as a white powder. mp 97–99 °C [lit. [60] 92–93 °C]; ^1^H-NMR (400 MHz, CDCl_3_) δ 9.56 (dd, 1H, *J* = 7.0, 1.2 Hz), 8.34 (m, 1H), 8.04 (s, 1H), 7.37 (m, 1H), 7.04 (td, 1H, *J* = 6.8, 1.2 Hz), 4.43 (q, 2H, *J* = 7.2 Hz), 3.97 (s, 3H), 1.47 (t, 3H, *J* = 7.2 Hz).

*1-Ethyl 3-methyl 7-(propylcarbamoyl)indolizine-1,3-dicarboxylate* (**17**). The reaction was performed with **5** (40 mg) and ethyl propiolate in methanol, following method C. The indolizine **17** was obtained in 66% yield (27 mg) as a white powder. mp 159–161°C; ^1^H-NMR (400 MHz, CDCl_3_) δ 9.52 (m, 1H), 8.64 (dd, 1H, *J* = 1.2 Hz, 2.0 Hz), 8.00 (s, 1H), 7.47 (dd, 1H, *J* = 2.0 Hz, 5.2 Hz), 6.38 (s br, 1H), 4.39 (q, 2H, *J* = 7.2 Hz), 3.94 (s, 3H), 3.46 (m, 2H), 1.68 (q, 2H, *J* = 7.2 Hz), 1.43 (t, 3H, *J* = 7.2 Hz), 1.01(t, 3H, *J* = 7.2 Hz); ^13^C-NMR (100 MHz, CDCl_3_) δ 165.3, 164.1, 161.3, 137.7, 131.4, 127.9, 124.7, 117.1, 115.6, 113.1, 107.3, 60.3, 51.7, 42.0, 22.9, 14.5, 11.5; HRMS (ESI) m/z: [M + H]^+^ calcd for C_17_H_21_N_2_O_5_ 333.1443, obsd 333.1445. 

*1-Ethyl 3-methyl 7-acetylindolizine-1,3-dicarboxylate* (**18**). The reaction was performed with **6** (100 mg) and ethyl propiolate in methanol, following method C. The indolizine **18** was obtained in 77% yield (40 mg) as a red powder. mp 151–152 °C; ^1^H-NMR (400 MHz, CDCl_3_) δ 9.55 (m, 1H), 8.98 (m, 1H), 8.07 (s, 1H), 7.57 (dd, 1H, *J* = 7.4 Hz, 1.9 Hz), 5.34 (s, 2H), 4.46 (q, 2H, *J* = 7.2 Hz), 4.00 (s, 3H), 2.75 (s, 3H), 1.49 (t, 3H, *J* = 7.2 Hz); ^13^C-NMR (100 MHz, CDCl_3_) δ 196.0, 163.8, 161.3, 137.5, 132.8, 127.6, 124.8, 121.4, 116.4, 111.8, 109.0, 60.4, 51.8, 26.1, 14.5; HRMS (ESI) *m/z*: [M + H]^+^ calcd for C_15_H_16_NO_5_ 290.1023, obsd. 290.1022, [M + Na]^+^ calcd for C_15_H_15_NNaO_5_ 312.0842, obsd. 312.0840.

*1-Ethyl 3-methyl 7-(trifluoromethyl)indolizine-1,3-dicarboxylate* (**19a**) and *1-methyl 3-methyl 7-(trifluoromethyl)indolizine-1,3-dicarboxylate* (**19b**). The reaction was performed with 4 (50 mg) and ethyl propiolate in methanol, following method C. The mixture of indolizines **19a** and **19b** (30/70 ratio) was obtained in 55% yield (27 mg) as a yellow powder. ^1^H-NMR (400 MHz, CDCl_3_) δ 9.60 (d, 1H, *J* = 7.2 Hz), 8.64 (s, 1H), 8.04 (s, 1H), 7.12 (dd, 1H, *J* = 7.2, 2.0 Hz), 4.40 (q, 2H, *J* = 7.2 Hz, CH_2_ of **19a**), 3.95 and 3.94 (2s, 2 × 3H, OMe of **19a** and **19b**), 1.42 (t, 3H, *J* = 7.2 Hz, CH_3_ of **19a**); ^13^C-NMR (100 MHz, CDCl_3_) δ 164.0, 163.5, 161.3, 136.8, 128.4,127.16, 125.7 (q, 225 Hz, CF_3_), 124.9, 124.6, 121.9, 117.5, 117.4, 116.1, 110.1, 107.9, 60.5, 51.8, 51.6, 14.5; HRMS (ESI) **19a** : *m/z* calcd for C_14_H_13_F_3_NO_4_ 316.0791, obsd 316.0796, **19b**: *m/z* calcd for C_13_H_11_F_3_NO_4_ 302.0635, obsd 302.0638.

*1-Ethyl 3-methyl 7-cyanoindolizine-1,3-dicarboxylate* (**20**). The reaction was performed with **7** (50 mg) and ethyl propiolate in methanol, following method C. The indolizine **20** was obtained in 81% yield (37 mg) as a white powder. mp 114–115 °C; ^1^H-NMR (400 MHz, CDCl_3_) δ 9.58 (dd, 1H, *J* = 7.2, 0.8 Hz), 8.74–8.75 (m, 1H), 8.06 (s, 1H), 7.07 (dd, 1H, *J* = 7.2, 2.0 Hz), 4.42 (q, 1H, *J* = 7.0 Hz), 3.98 (s, 3H), 1.44 (t, 3H, *J* = 7.2 Hz); ^13^C-NMR (100 MHz, CDCl_3_) δ 163.2, 161.1, 136.0, 128.2, 125.9, 125.1, 117.5, 114.1, 109.0, 108.0, 60.7, 52.0, 14.5; HRMS (ESI) *m/z*: [M + H]^+^ calcd for C_14_H_13_N_2_O_4_ 273.0869, obsd 273.0870.

*1,3-Diethyl 7-acetylindolizine-1,3-dicarboxylate* (**22**) [61]. The reaction was performed with **12** (100 mg) and ethyl propiolate, following method D. The indolizine **22** was obtained in 12% yield (9 mg) as a white powder. mp 125–127 °C. ^1^H-NMR (400 MHz, DMSO-*d*_6_) *δ* 9.47 (dd, 1H, *J* = 0.8, 7.4 Hz), 8.81 (d, 1H, *J* = 0.8 Hz), 7.90 (s, 1H), 7.62 (dd, 1H, *J* = 1.9, 7.4 Hz), 4.35–4.40 (m, 4H), 2.69 (s, 3H), 1.35–1.41 (m, 6H).

*1,3-Diethyl 7-cyanoindolizine-1,3-dicarboxylate* (**23**). The reaction was performed with **13** (15 mg) and ethyl propiolate, following method D. The indolizine **23** was obtained in 40% yield (4.9 mg) as a white powder. mp 103–104 °C; ^1^H-NMR (400 MHz, CDCl_3_) δ 9.64 (dd, 1H, *J* = 0.8, 7.2 Hz), 8.78 (s, 1H), 8.11 (s, 1H), 7.11 (dd, 1H, *J* = 1.6, 7.2 Hz), 4.45–4.50 (m, 4H), 1.46–1.51 (m, 6H); ^13^C-NMR (100 MHz, CDCl_3_) δ 163.2, 161.1, 136.1, 128.2, 125.8, 125.0, 117.5, 116.9, 114.1, 109.0, 108.0, 61.4, 60.7, 52.0, 14.5 (identical to the commercial compound).

*1-Ethyl 3-benzoyl-7-cyanoindolizine-1-carboxylate* (**24**). The reaction was performed with **8** (50 mg) and ethyl propiolate in methanol, following method C. The indolizine **24** was obtained in 50% yield (22.4 mg) as an orange powder. mp 136–137 °C; ^1^H-NMR (400 MHz, CDCl_3_) δ 9.99 (d, 1H, *J* = 6.8 Hz), 8.83 (s, 1H), 7.95 (s, 1H), 7.87 (d, 2H, *J* = 6.8 Hz), 7.67 (d, 1H, *J* = 6.4 Hz), 7.60 (d, 2H, *J* = 7.2 Hz), 7.20 (d, 1H, *J* = 6.8 Hz), 4.47 (q, 2H*, J =* 7.2 Hz), 1.47 (t, 3H, *J* = 7.2 Hz); ^13^C-NMR (100 MHz, CDCl_3_) δ 186.1, 163.2, 138.9, 136.9, 132.3, 129.3, 129.1, 129.0, 128.7, 128.4, 125.6, 124.2, 117.3, 114.8, 109.7, 109.4, 60.8, 14.53; HRMS (ESI) *m/z* calcd for C_19_H_15_N_2_O_3_ 319.1077, obsd 319.1081.

*1-Ethyl 3-(4-nitrobenzoyl)-7-cyanoindolizine-1-carboxylate* (**25**). The reaction was performed with **9** (50 mg) and ethyl propiolate in methanol, following method C. The indolizine **25** was obtained in 67% yield (29.6 mg) as a yellow powder. mp 143–144 °C; ^1^H-NMR (400 MHz, CDCl_3_) δ 9.92 (d, 1H, *J* = 7.2 Hz), 8.75 (s, 1H), 8.34 (d, 2H, *J* = 8.5 Hz), 7.90 (d, 2H, *J* = 8.4 Hz), 7.76 (s, 1H), 7.17 (m, 1H), 4.35 (q, 2H, *J* = 7.1 Hz), 1.35 (t, 3H, *J* = 7.1 Hz); ^13^C-NMR (100 MHz, CDCl_3_) δ 183.9, 163.0, 150.0, 144.3, 137.7, 130.1, 129.9, 129.6, 129.3, 125.9, 124.1, 123.9, 123.6, 117.2, 115.7, 110.9, 110.2, 61.2, 14.7; HRMS (ESI) *m/z*: [M + H]^+^ calcd for C_19_H_14_N_3_O_5_ 364.0927, obsd 364.0928.

*1-Ethyl 7-cyano-3-(N-propylcarbamoyl)indolizine-1-carboxylate* (**26**). The reaction was performed with **10** (20 mg) and ethyl propiolate in methanol, following method C. The indolizine **26** was obtained in 62% yield (15.6 mg) as a white powder. mp 170–171 °C; ^1^H-NMR (400 MHz, CDCl_3_) δ 9.83 (dd, 1H, *J* = 7.4, 1.0 Hz), 8.75 (dd, 1H, *J* = 1.8, 1.0 Hz), 7.80 (s, 1H), 7.08 (dd, 1H, *J* = 7.4, 1.8 Hz), 6.17 (br s, 1H), 4.51 (q, 2H, *J* = 7.2 Hz), 3.40–3.55 (m, 2H), 1.72–1.79 (m, 2H), 1.53 (t, 3H, *J* = 7.2 Hz), 1.10 (t, 3H, *J* = 7.2 Hz); ^13^C-NMR (100 MHz, CDCl_3_) δ 163.4, 160.7, 135.1, 128.7, 125.8, 119.9, 119.2, 117.7, 113.4, 108.2, 107.3, 60.7, 41.3, 23.0, 14.6, 11.5; HRMS (ESI) *m/z*: [M + H]^+^ calcd for C_16_H_18_N_3_O_3_ 300.1342, obsd 300.1348. 

*Methyl 1-(N-benzylcarbamoyl)-7-cyanoindolizine-3-carboxylate* (**28**). The reaction was performed with propiolamide **27** and pyridinium salt **7** (50 mg) in methanol, following method C. The indolizine **28** was obtained in 40% yield (26 mg) as a white powder. mp 196–199 °C; ^1^H-NMR (400 MHz, CDCl_3_) δ 9.56 (dd, 1H, *J* = 7.4, 1.2 Hz), 9.07 (dd, 1H, *J* = 1.8, 1.2 Hz), 7.77 (s, 1H), 7.42–7.44 (m, 4H), 7.36–7.39 (m, 1H), 7.10 (dd, 1H, *J* = 7.4, 1.8 Hz), 6.29 (br s, 1H), 4.72 (d, 2H, *J* = 5.6 Hz), 3.99 (s, 3H); ^13^C-NMR (100 MHz, CDCl_3_) δ 162.9, 160.9, 138.1, 136.0, 128.9 (2C), 128.0 (2C), 127.8, 127.7, 126.8, 120.1, 117.5, 116.3, 114.2, 111.5, 107.5, 51.9, 43.7; HRMS (ESI) *m/z*: [M + H]^+^ calcd for C_19_H_16_N_3_O_3_ 334.1186, obsd 334.1183, [M + Na]^+^ calcd for C_19_H_15_N_3_O_3_ 356.1006, obsd. 356.1007.

### 4.4. pKa Determination

Potentiometric measurements were performed in a jacketed cell thermostated at 25.0 °C, kept under an inert atmosphere of purified argon, using an automatic titrator (Metrohm, DMS Titrino 716, Herisau, Switzerland) connected to a microcomputer. The free hydrogen concentrations were measured with a glass-Ag/AgCl combined electrode (Metrohm) filled with 0.1 M NaCl. The electrode was calibrated with three standard buffers at pH 4, 7, and 10. NaCl was employed as supporting electrolyte to maintain the ionic strength at 0.10 M.

Samples of 0.2 mmol of pyridinium salts were dissolved in 20 mL of fresly prepared 0.1 M NaClO_4_. Aliquot of 10 mL were titrated with 0.02 M NaOH. A minimum of three sets of data was used in each case. Equilibrium constants and species distribution diagrams were calculated by using the program HYPERQUAD 2003.

## Figures and Tables

**Table 1:** Structures, yields, pKa values, and proton NMR data of the pyridinium salts. 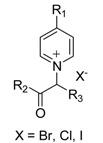

Salt	R_1_	R_2_	R_3_	Yields %	R_1_ Hammett Cste	pKa ^a^	^1^H-NMR ^b^ δ H2/H6	^1^H-NMR ^b^ δ H3/H5	^1^H-NMR ^b^ δ CH_2_/CH
**1**	H	OMe	H	71	0	8.30	9.01	8.23	5.66
**2**	NH_2_	OMe	H	83	−0.66	8.84	8.09	6.92	5.12
**3**	NHAc	OMe	H	81	0.06	8.74	8.74	8.14	5.51
**4**	CF_3_	OMe	H	54	0.54	8.25	9.39	8.66	5.83
**5**	CONHPr	OMe	H	80	0.36	8.24	9.19	8.52	5.75
**6**	COCH_3_	OMe	H	64	0.50	8.04	9.35	8.63	5.88
**7**	CN	OMe	H	60	0.66	8.16	9.29	8.64	5.75
**8**	CN	Ph	H	70		8.11	9.30	8.85	6.61
**9**	CN	Ph-NO_2_	H	60		7.07	9.26	8.83	6.60
**10**	CN	NHC_3_H_7_	H	60		nd	9.24	8.59	5.60
**11**	H	OEt	CO_2_Et	89		5.51	9.20	8.28	Not obs.
**12**	COCH_3_	OEt	CO_2_Et	99		5.04	9.36	8.61	Not obs.
**13**	CN	OEt	CO_2_Et	40		nd ^c^	9.44	8.66	Not obs.

^a^ The pKa values were determined by potentiometry of 10^−3^ M solution of the salt in 0.1 M NaClO_4_. The measurements were made in triplicate; ^b^ The NMR spectra were recorded in CD_3_OD; ^c^ Non-reproducible data.

**Table 2:** Influence of the pyridine substituent R_1_ on the yields of indolizines ^a^. 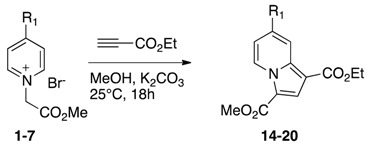

Entry	Pyridinium Salt	R_1_	Hammett Constant	Indolizine	Yield (%)
1	**1**	**H**	0	**14**	59
2	**2**	**NH_2_**	−0.66	**15**	0
3	**3**	**NHAc**	0.06	**16**	0
4	**5**	**CONHPr**	0.36	**17**	66
5	**6**	**COCH_3_**	0.50	**18**	77
6	**4**	**CF_3_**	0.54	**19**	55 ^b^
7	**7**	**CN**	0.66	**20**	81

^a^ The pyridinium salts (1 eq.) and ethyl propiolate (1.5 eq.) were dissolved in MeOH. K_2_CO_3_ (1 eq.) was then added to generate the ylides and the resulting solutions were stirred 18h at room temperature. ^b^ Mixtures of **19** and of the corresponding 1,3-dimethyl carboxylate analogue were obtained in a non-reproducible ratio.

**Table 3:** Influence of the nature of the solvent and of the base on the yields of indolizines **18** and **20** from pyridinium salts **6** and **7**. 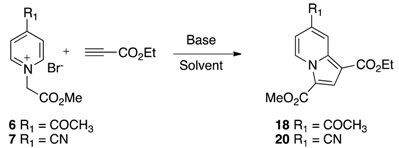

Entry	R_1_	Base (1 eq.)	Solvent	T °C	Time (h)	Yield (%)
1	COCH_3_	K_2_CO_3_	MeOH	25	1	50
2	COCH_3_	K_2_CO_3_	MeOH	25	18	77
3	COCH_3_	NH_4_OH	MeOH	25	1	45
4	COCH_3_	NEt_3_	MeOH	25	1	38
5	COCH_3_	K_2_CO_3_	DMF	25	1	88
6	COCH_3_	NH_4_OH	DMF	25	1	44
7	COCH_3_	NEt_3_	DMF	25	1	45
8	COCH_3_	K_2_CO_3_	DMF	25	5	>90
9	COCH_3_	NH_4_OH	DMF	25	5	48
10	COCH_3_	NEt_3_	DMF	25	5	48
11	CN	K_2_CO_3_	MeOH	25	5	45
12	CN	K_2_CO_3_	MeOH	25	18	81
13	CN	K_2_CO_3_	DMF	25	5	70
14	CN	K_2_CO_3_	MeOH	40	18	38
15	CN	pH 7.5 Phosphate buffer	H_2_O	25	18	24

**Table 4:** Formation of indolizine from diethyl dicarboxylate substituted pyridinium **11**–**13** in pH 7.5 buffer solutions. 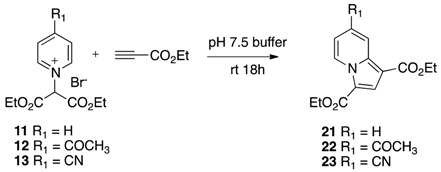

Entry	R_1_	Base	Solvent	T °C	Indolizine	Yield (%)
1	H	pH 7.5 Tris buffer	H_2_O	40	**21**	0
2	COCH_3_	pH 7.5 Tris buffer	H_2_O	40	**22**	12
3	CN	pH 7.5 Tris buffer	H_2_O	40	**23**	40
4	CN	pH 7.5 Tris buffer	H_2_O	25	**23**	63
5	CN	pH 7.5 phosphate buffer	H_2_O	25	**23**	42
6	CN	K_2_CO_3_	MeOH	25	**23**	0
7	CN	K_2_CO_3_	DMF	25	**23**	93

The mixtures of pyridinium salts (1 eq.) and ethyl propiolate (1.5 eq.) were stirred at the chosen temperatures for 18 h. Results are given as yields of isolated indolizines.

**Table 5:** Effect of the nature of R_2_ group on the yield of 7-cyanoquinazolines. 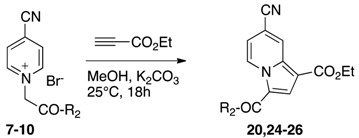

Entry	Pyridinium Salt	R_2_	Indolizine	Yield (%)
1	**7**	**OMe**	**20**	81
2	**8**	**Ph**	**24**	50
3	**9**	**C_6_H_4_NO_2_**	**25**	67
4	**10**	**NHC_3_H_7_**	**26**	62

The pyridinium salts and 1.5 equivalents of ethyl propiolate were stirred in methanol in the presence of K_2_CO_3_ (1 eq.) for 18 h at room temperature.

**Table 6:** Reaction of 4-cyanopyridinium salt **7** with various dipolarophiles.

Entry	Dipolarophile	Indolizine (Yield)
1	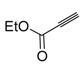	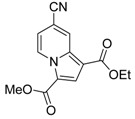 **20** (81%)
2	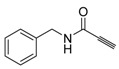 **27**	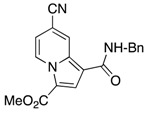 **28** (40%)
3	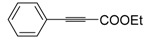 **29**	Complex mixture
4	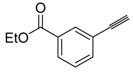 **30**	(0%)
5	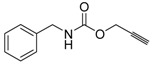 **31**	(0%)

Stoichiometric amounts of 4-cyanopyridinium **7**, the chosen dipolarophile and K_2_CO_3_ in methanol were stirred at room temperature for 18 h. Yields are given after purification of the indolizines by column chromatography (elution EtOAc/cyclohexane).

## Supplementary Materials

Supplementary materials can be accessed at: http://www.mdpi.com/1420-3049/21/3/332/s1.

## Abbreviations

The following abbreviations are used in this manuscript:

DMF: *N,N*-dimethylformamide
DMSO-D_6_: deuterated dimethylsulfoxide
TLC: thin-layer chromatography
TRIS: Tris(hydroxymethyl)aminomethane
