# Supplementary material for: Investigation of the Pyridinium Ylide—Alkyne Cycloaddition as a Fluorogenic Coupling Reaction
Source: Molecules. 2016 Mar 10;21(3):332. doi: 10.3390/molecules21030332 (PMC6273055; doi:10.3390/molecules21030332)
Supplement: Supplementary file 1 [file molecules-21-00332-s001.pdf]

Supplementary Materials: Investigation of the Pyridinium Ylide-Alkyne Cycloaddition as Fluorogenic Coupling Reaction

Simon Bonte, Ioana Ghinea, Rodica Dinica, Isabelle Baussanne, Martine Demeunynck

Supplementary material

Synthesis:

Table 1. Preparation of the heterocyclic salt precursor. Page S2

NMR and HPLC Chromatograms: Pages S3–S26

Table S1. Preparation of pyridinium salts and analogs.

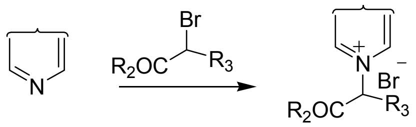

Starting Material	Acylating Agent	Method	Pyridinium Salt	Yield (%)
pyridine	$BrCH_2CO_2Me$	A	**1**	71
4-aminopyridine	$BrCH_2CO_2Me$	A	**2**	83
4-N-acetylamino pyridine	$BrCH_2CO_2Me$	A	**3**	81
4-trifluoromethyl pyridine	$BrCH_2CO_2Me$	A	**4**	54
4-propylcarbamoyl pyridine	$BrCH_2CO_2Me$	A	**5**	80
4-acetylpyridine	$BrCH_2CO_2Me$	A	**6**	64
4-cyanopyridine	$BrCH_2CO_2Me$	A	**7**	60
4-cyanopyridine	$BrCH_2COPh$	A	**8**	70
4-cyanopyridine	$BrCH_2COPhNO_2$	A	**9**	60
4-cyanopyridine	$ClCH_2CONHC_3H_7$	A	**10**	60
pyridine	$BrCH(CO_2CH_2CH_3)_2$	B	**11**	89
4-acetylpyridine	$BrCH(CO_2CH_2CH_3)_2$	B	**12**	99
4-cyanopyridine	$BrCH(CO_2CH_2CH_3)_2$	B	**13**	40

Method A: the alkylation was performed under ultrasound irradiation (3–5 h) in acetone, using 1.5 eq. of alkylating agent. The final temperature of the bath was kept below 50 °C; Method B: the reactions were performed at rt using diethyl 2-iodomalonate in large excess in acetone

1-(2-Methoxy-2-oxoethyl)pyridinium bromide (1):

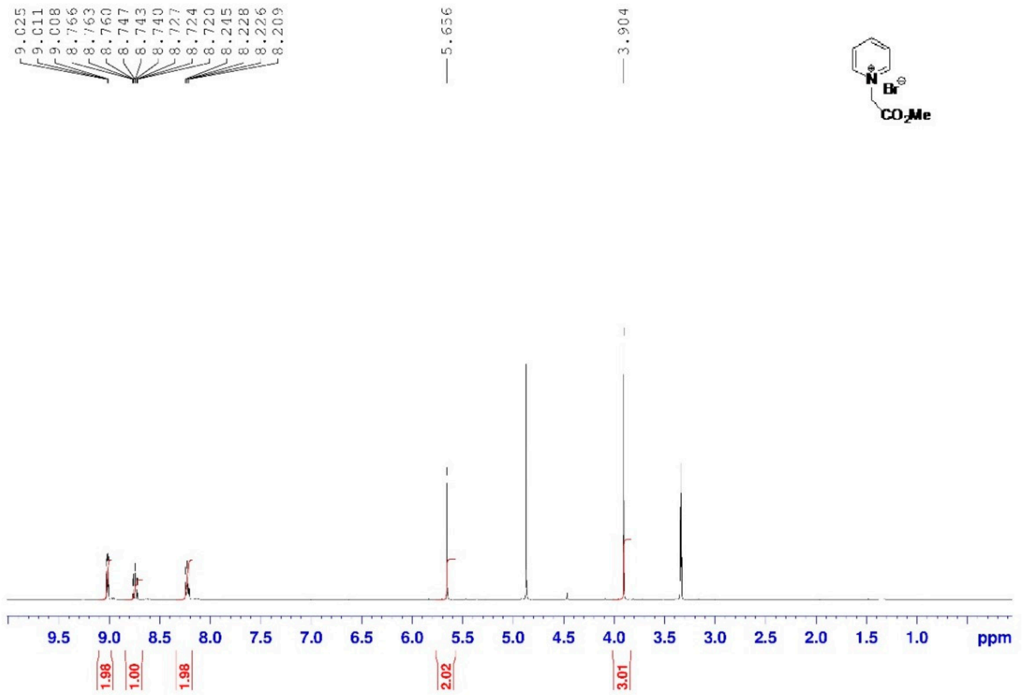

4-Amino-1-(2-methoxy-2-oxoethyl)pyridinium bromide (2)

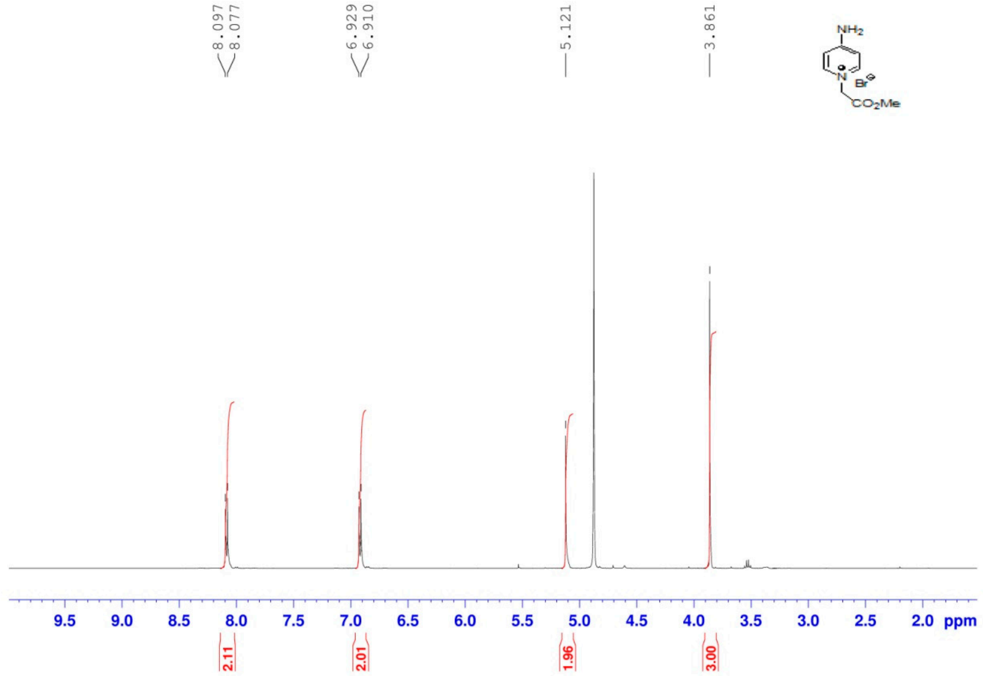

4-Acetamido-1-(2-methoxy-2-oxoethyl)pyridinium bromide (3)

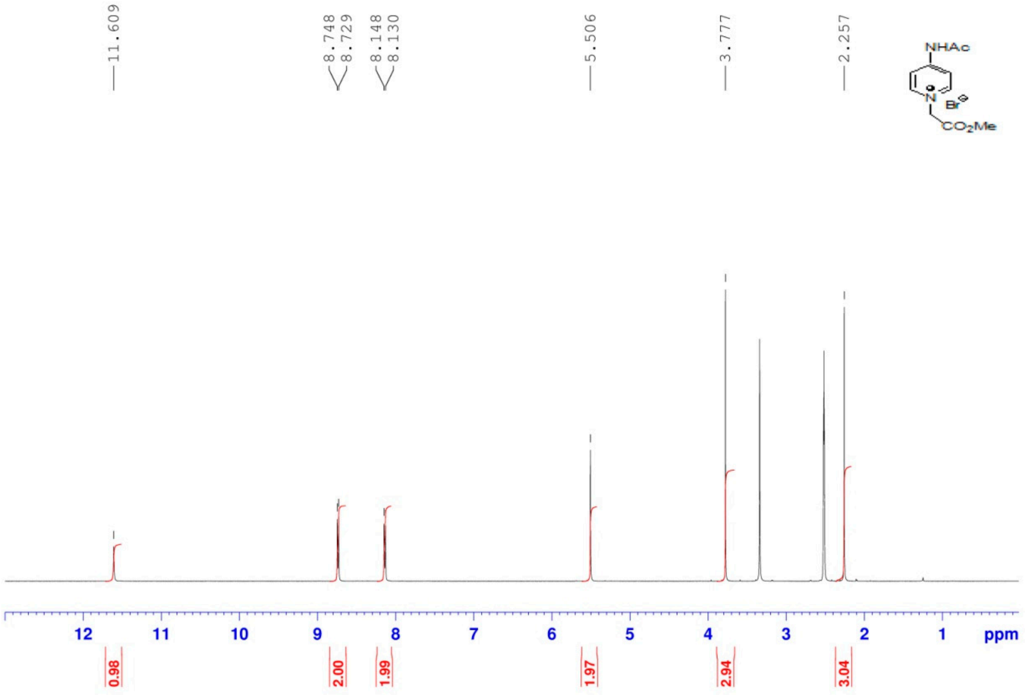

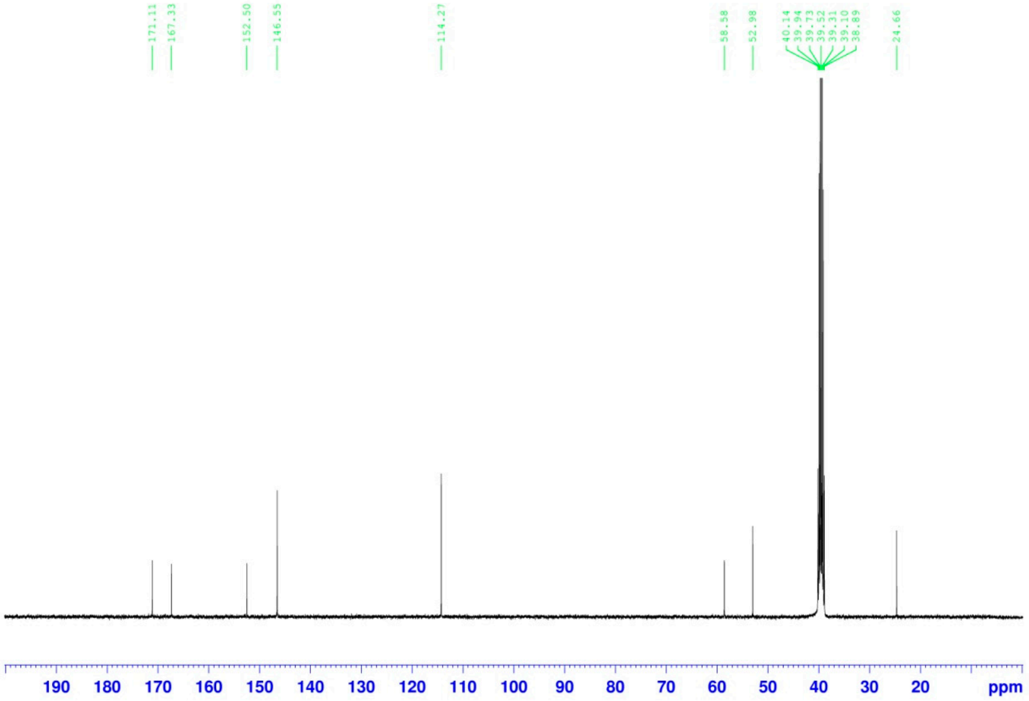

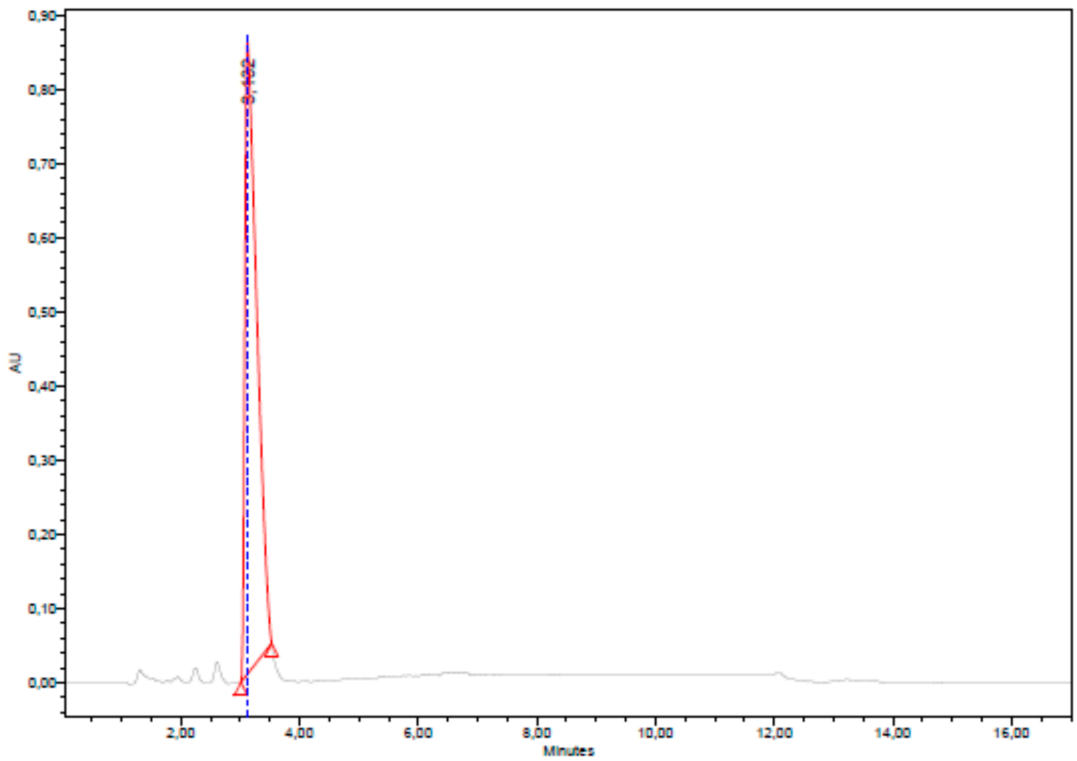

4-Trifluoromethyl-1-(2-methoxy-2-oxoethyl)pyridinium bromide (4)

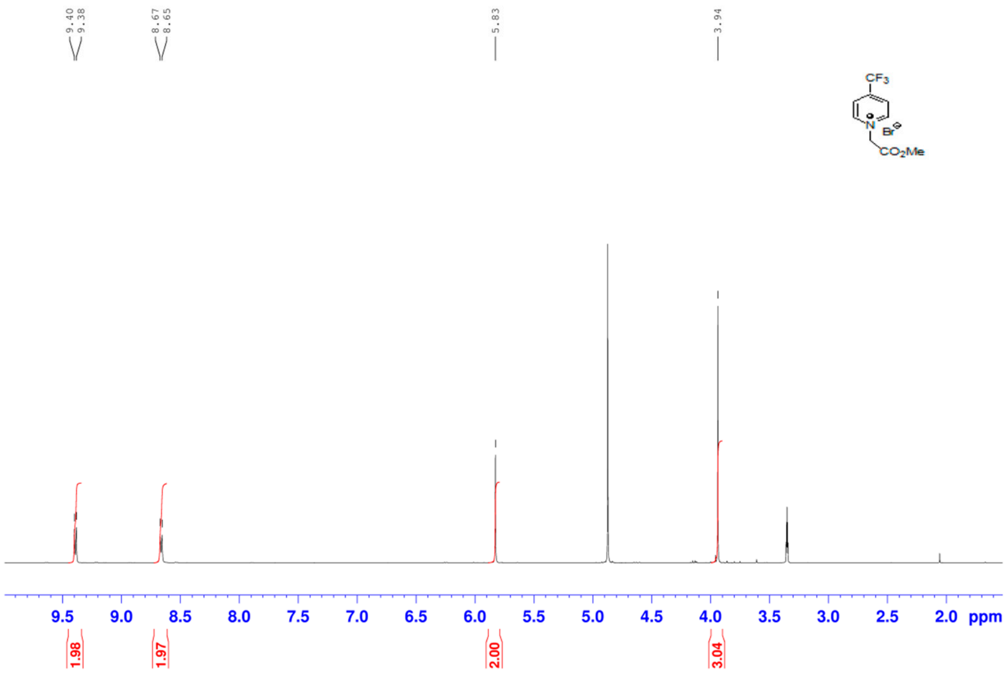

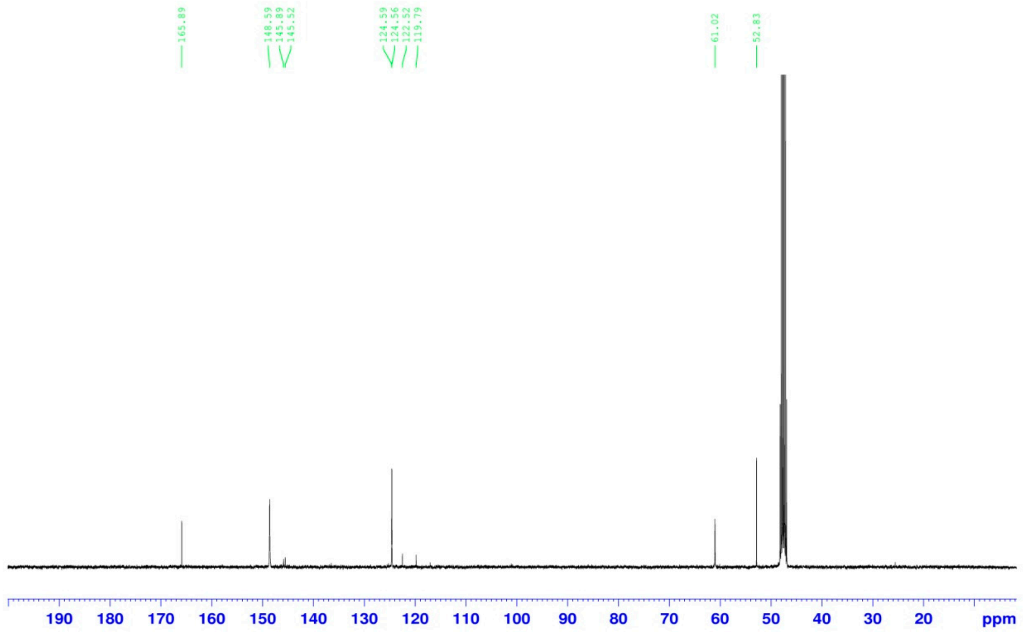

4-(*N*-Propylcarbamoyl)-1-(2-methoxy-2-oxoethyl)pyridinium bromide (5)

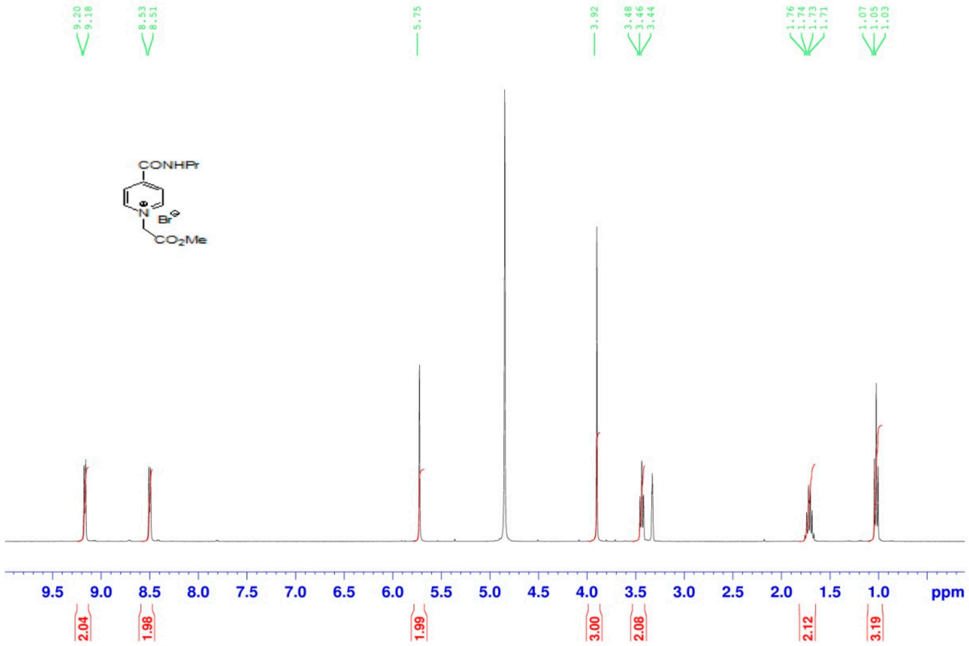

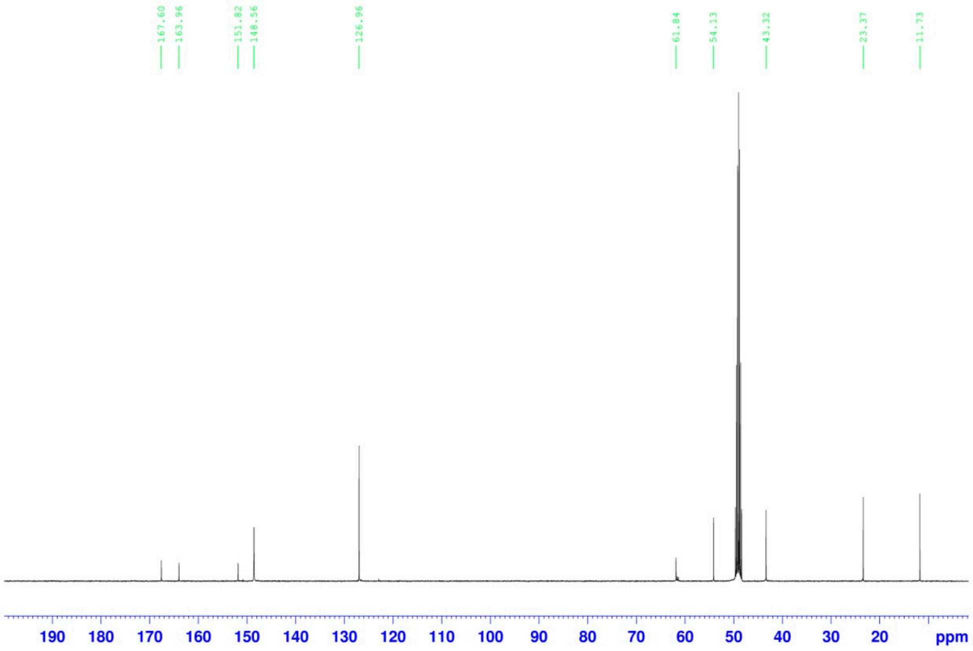

4-Acetyl-1-(2-methoxy-2-oxoethyl)pyridinium bromide (6)

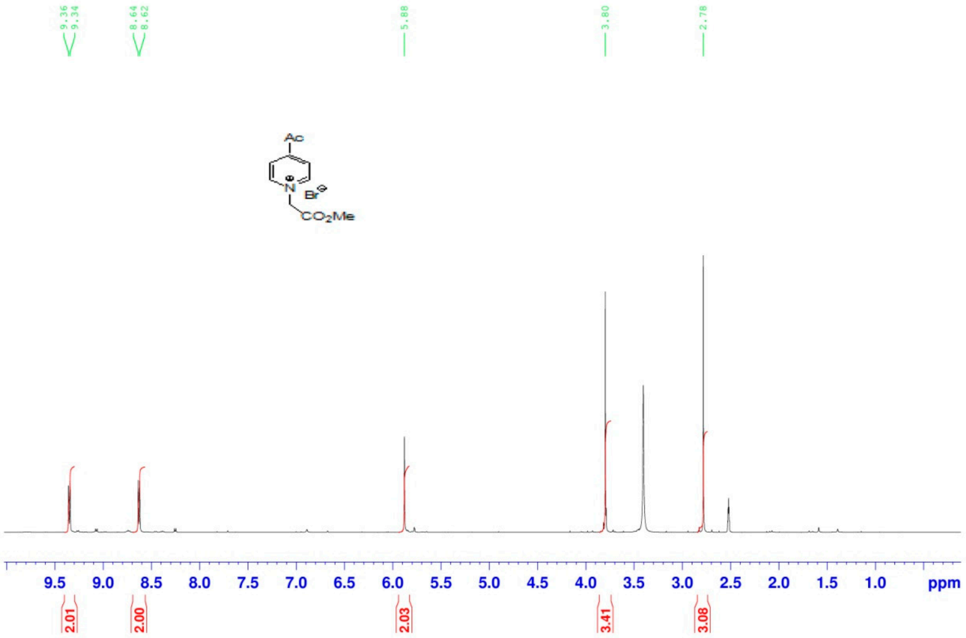

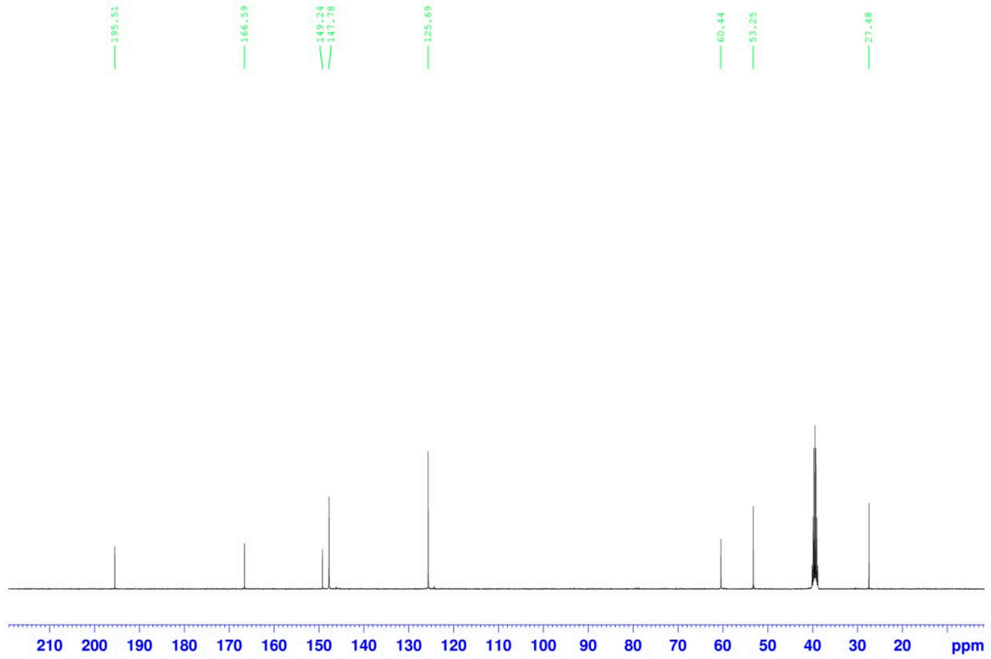
195.51
166.59
149.24
147.78
125.69
60.44
53.25
27.48
210 200 190 180 170 160 150 140 130 120 110 100 90 80 70 60 50 40 30 20
ppm

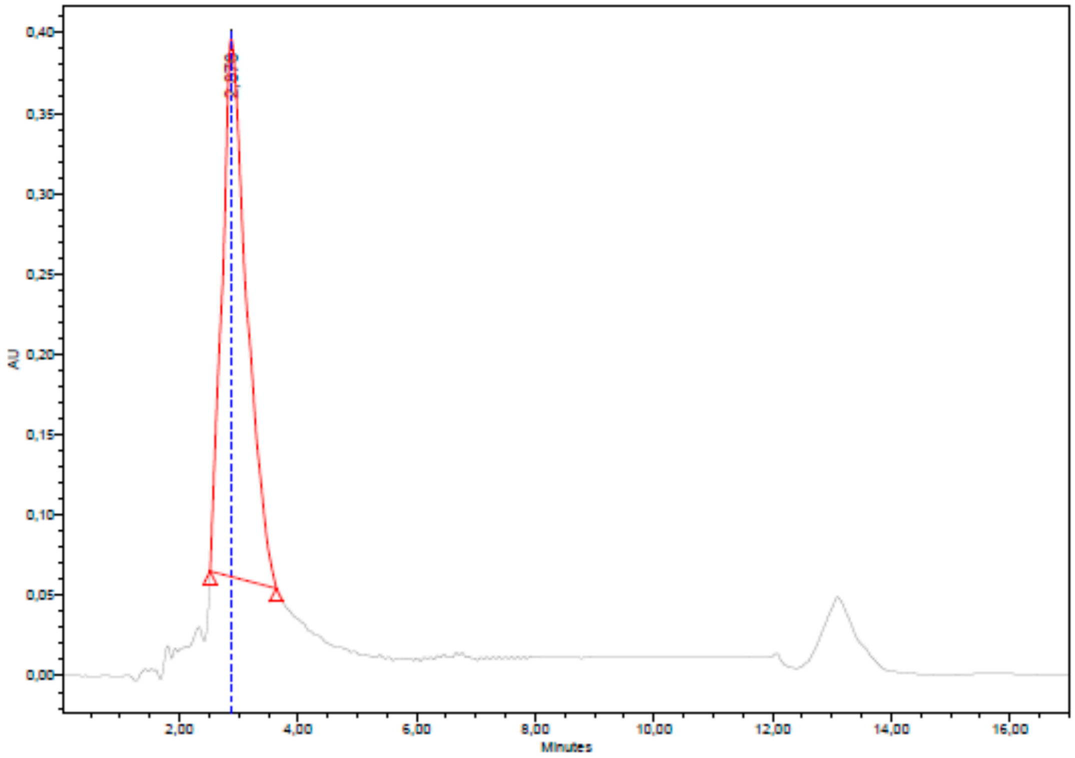
AU
0,40
0,35
0,30
0,25
0,20
0,15
0,10
0,05
0,00
2,00 4,00 6,00 8,00 10,00 12,00 14,00 16,00
Minutes

4-Cyano-1-(2-methoxy-2-oxoethyl)pyridinium bromide (7)

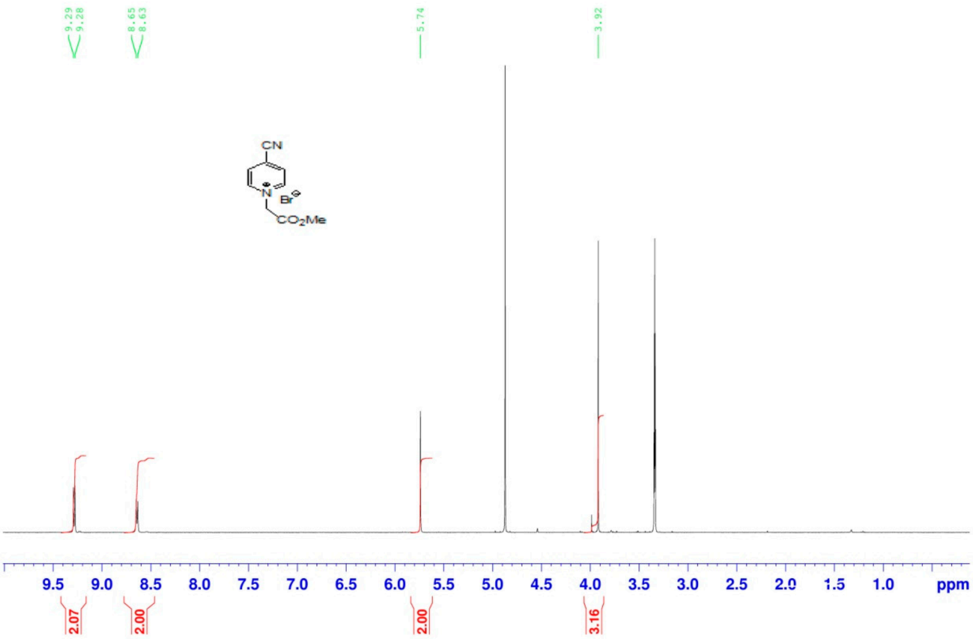

4-Cyano-1-(2-oxo-2-phenylethyl)pyridinium iodide (8)

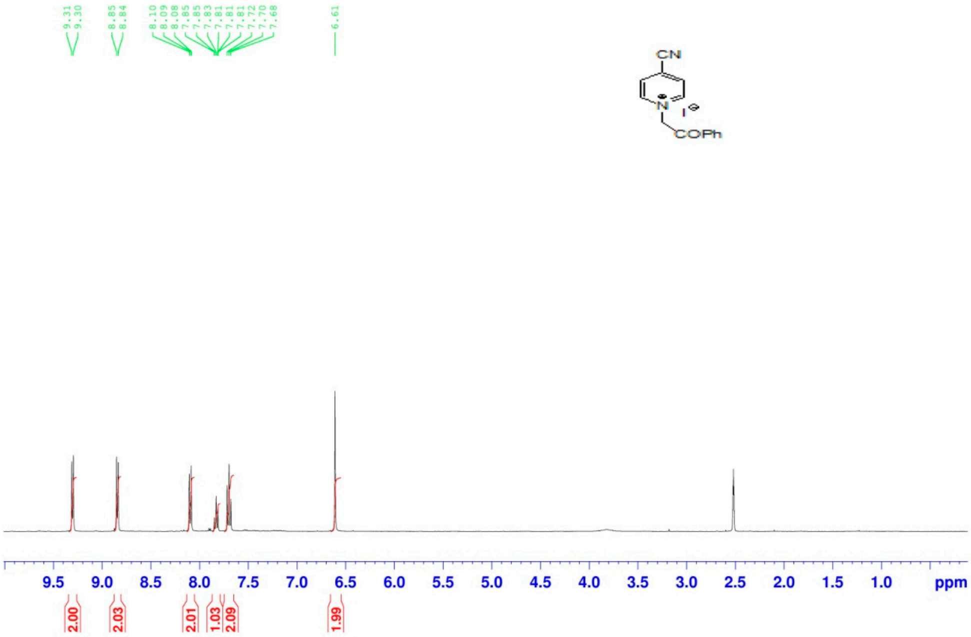

4-Cyano-1-(2-oxo-2-(para-nitrophenyl)ethyl)pyridinium iodide (9)

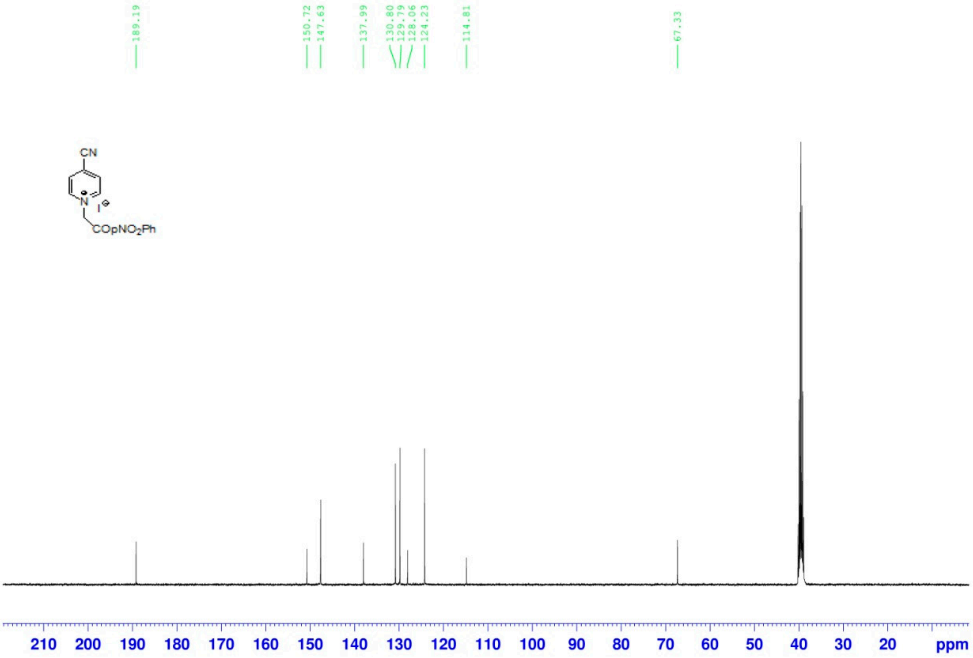

4-Cyano-1-[(*N*-propylcarbamoyl)methyl]pyridinium bromide (10)

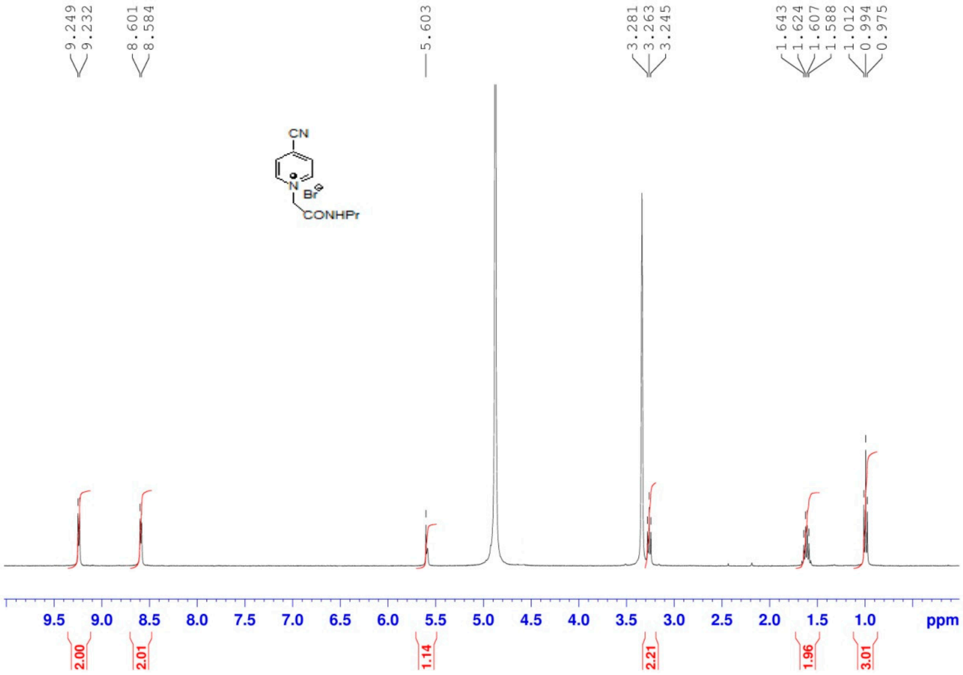

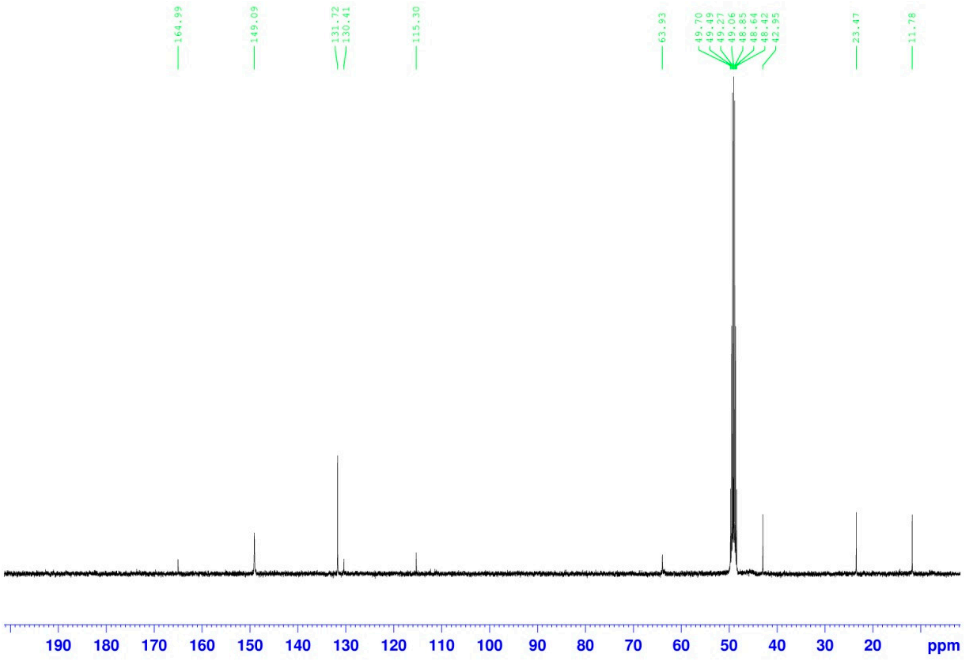
164.99
149.09
131.72
130.41
115.30
63.93
49.70
49.49
49.27
49.06
48.85
48.64
48.42
42.95
23.47
11.78
190
180
170
160
150
140
130
120
110
100
90
80
70
60
50
40
30
20
ppm

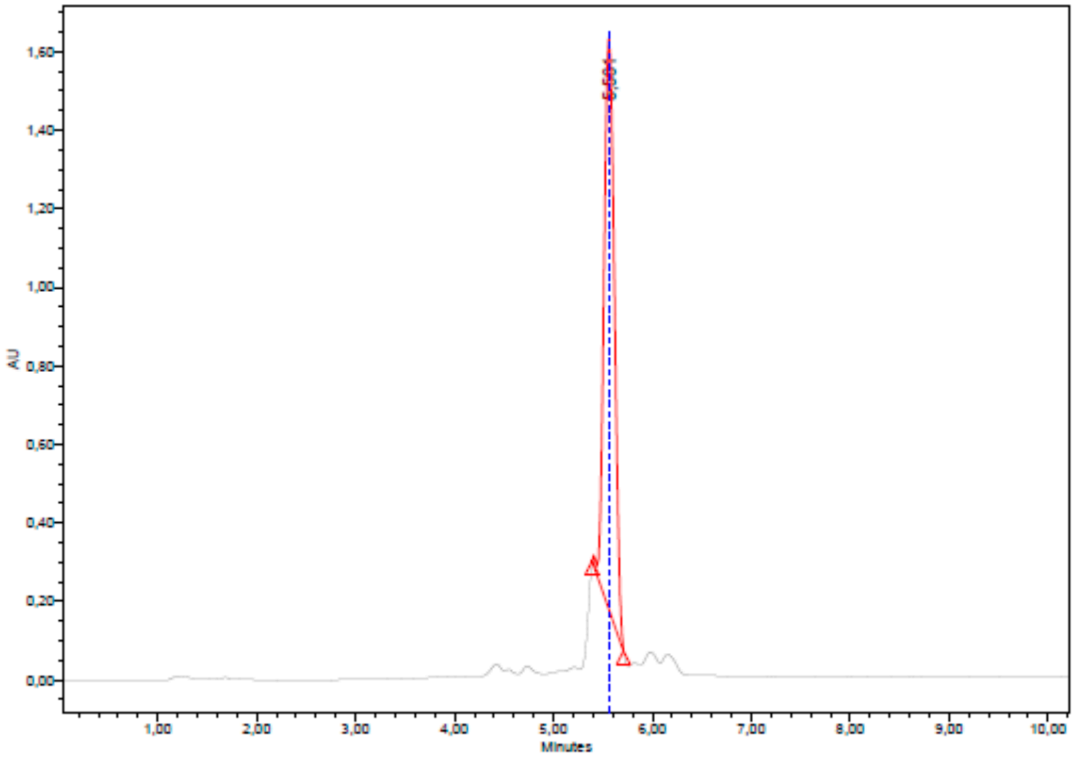
AU
1,60
1,40
1,20
1,00
0,80
0,60
0,40
0,20
0,00
1,00
2,00
3,00
4,00
5,00
6,00
7,00
8,00
9,00
10,00
Minutes

1-(1,3-Diethoxy-1,3-dioxopropan-2-yl)pyridinium iodide (11)

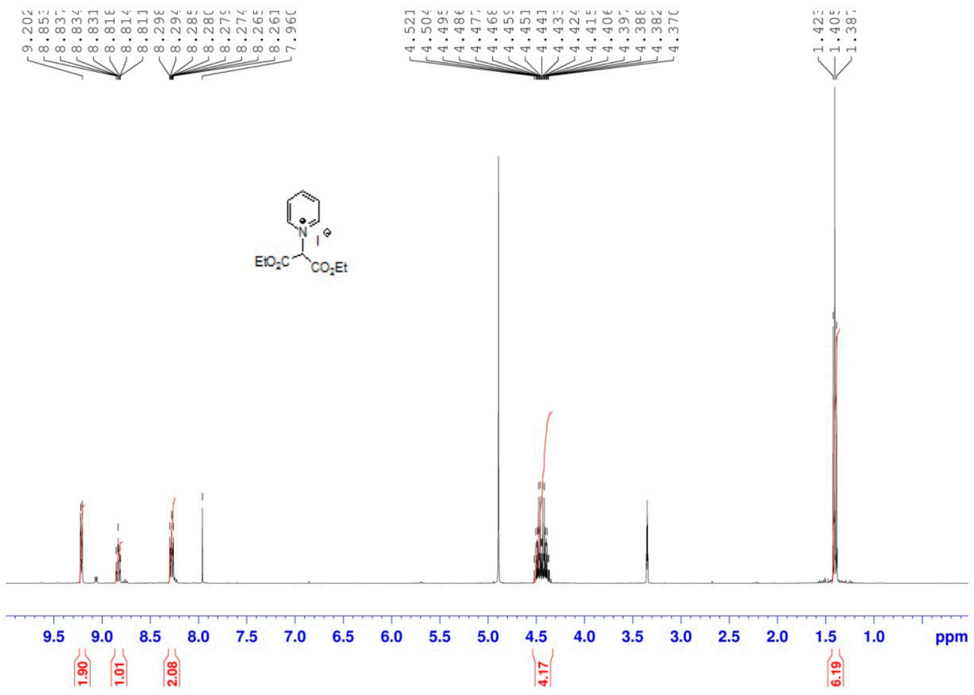

4-Acetyl-1-(1,3-diethoxy-1,3-dioxopropan-2-yl)pyridinium iodide (12)

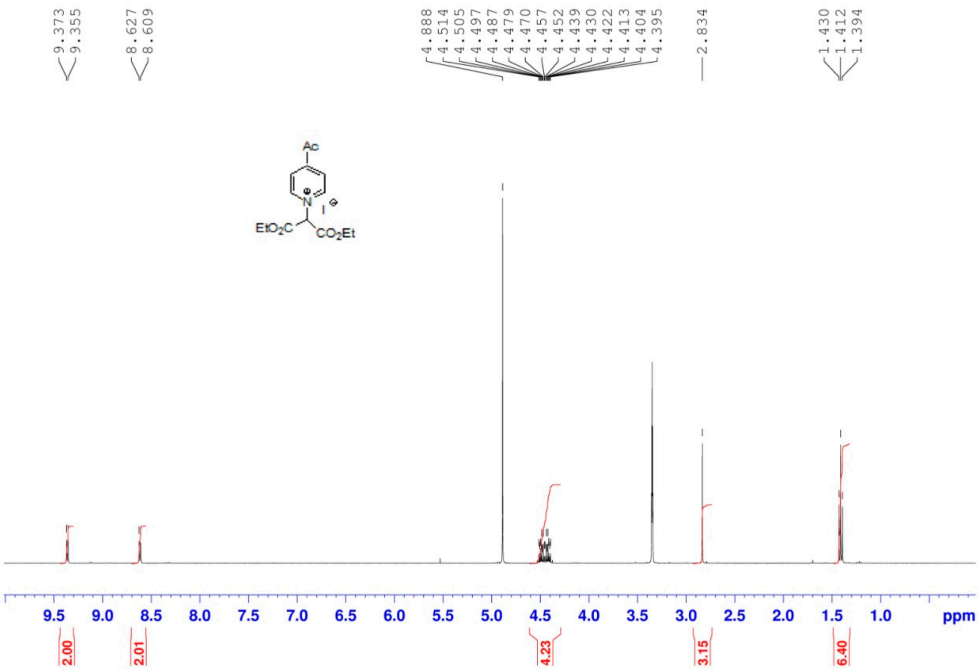

4-Cyano-1-(1,3-diethoxy-1,3-dioxopropan-2-yl)pyridinium iodide (13)

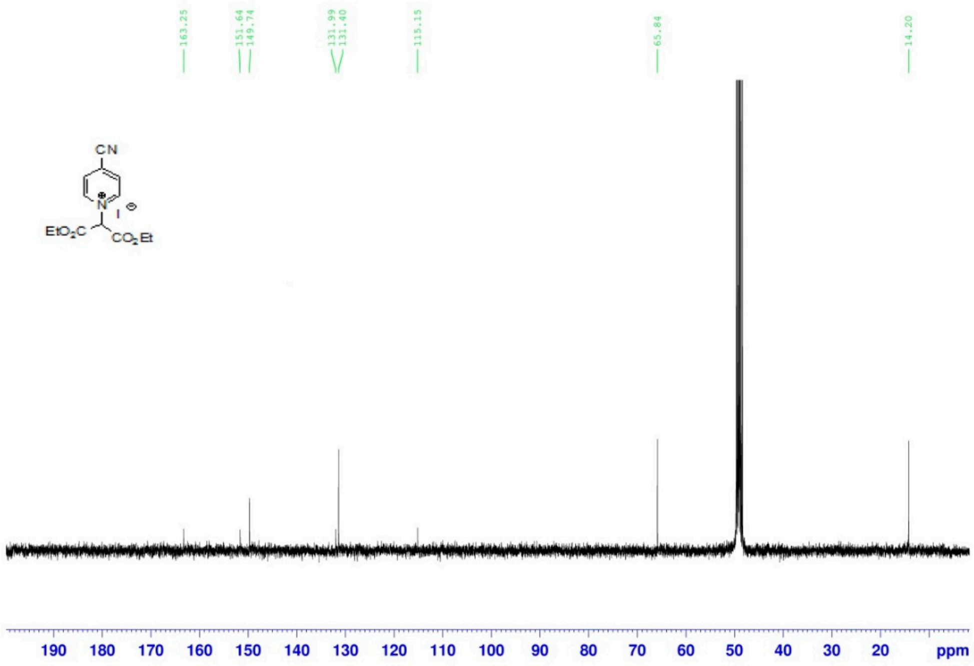

1-Ethyl 3-methyl indolizine-1,3-dicarboxylate (14)

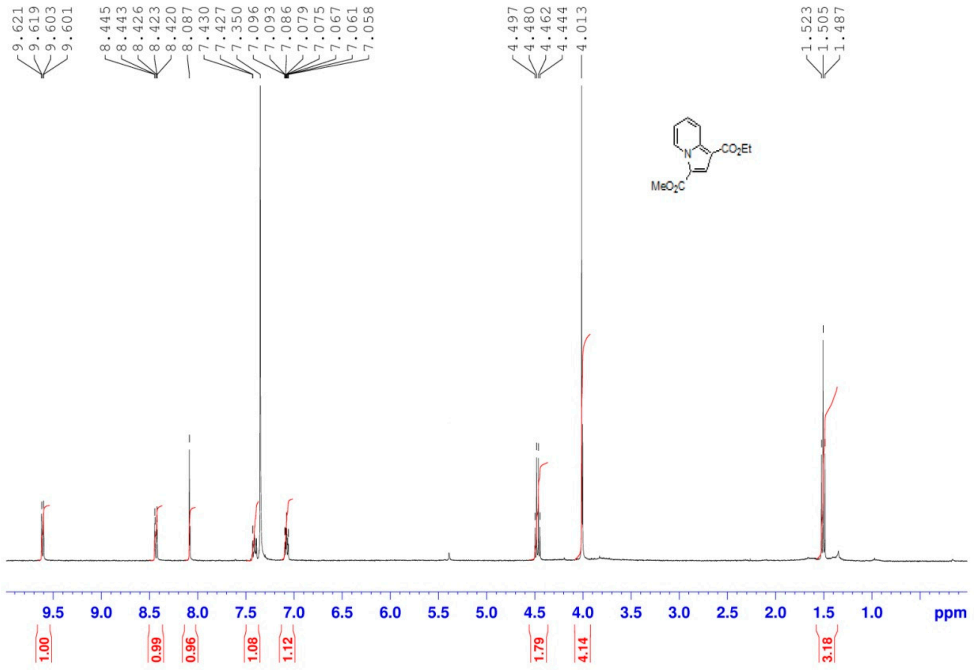

1-Ethyl 3-methyl 7-(propylcarbamoyl)indolizine-1,3-dicarboxylate (17)

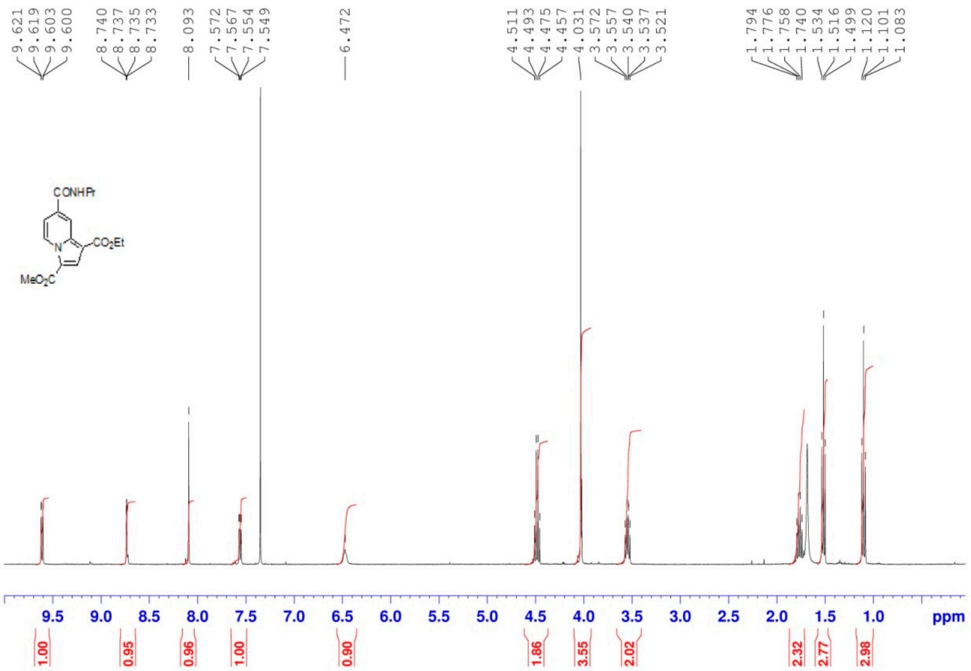

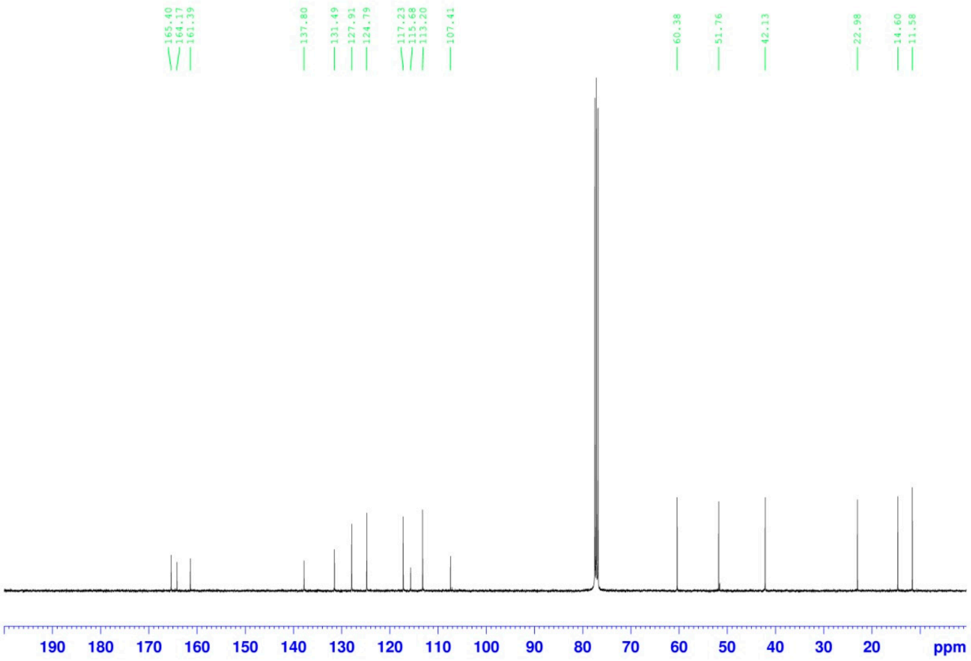

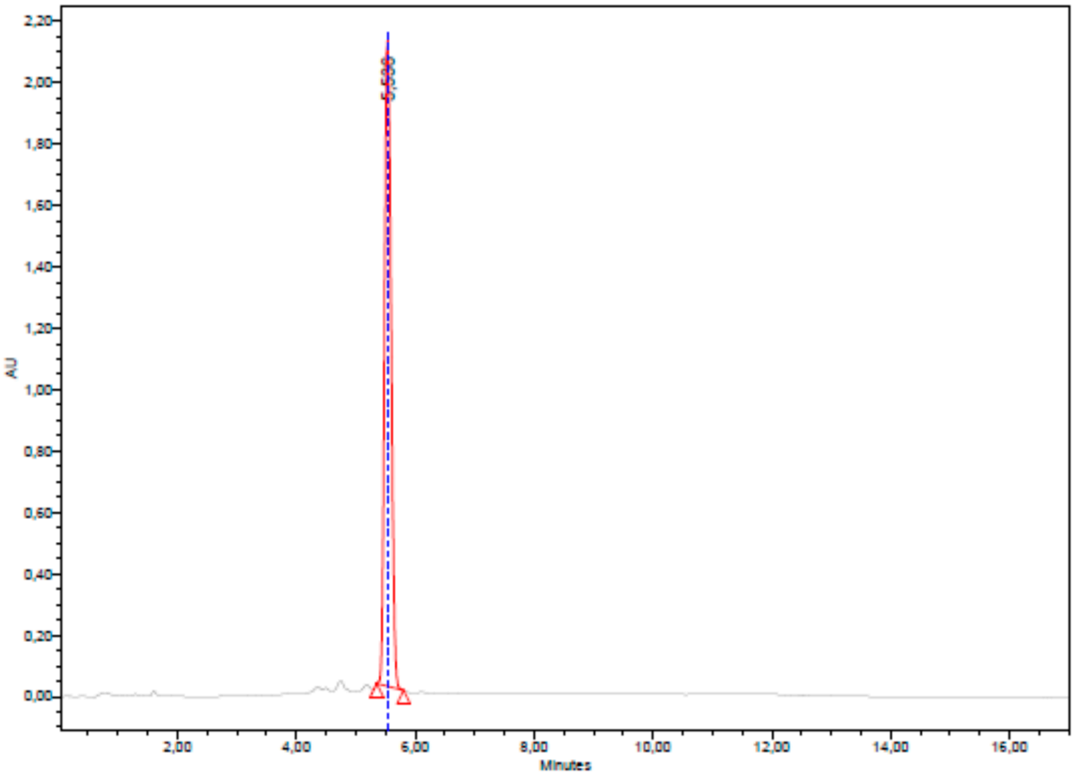

1-Ethyl 3-methyl 7-acetylindolizine-1,3-dicarboxylate (18)

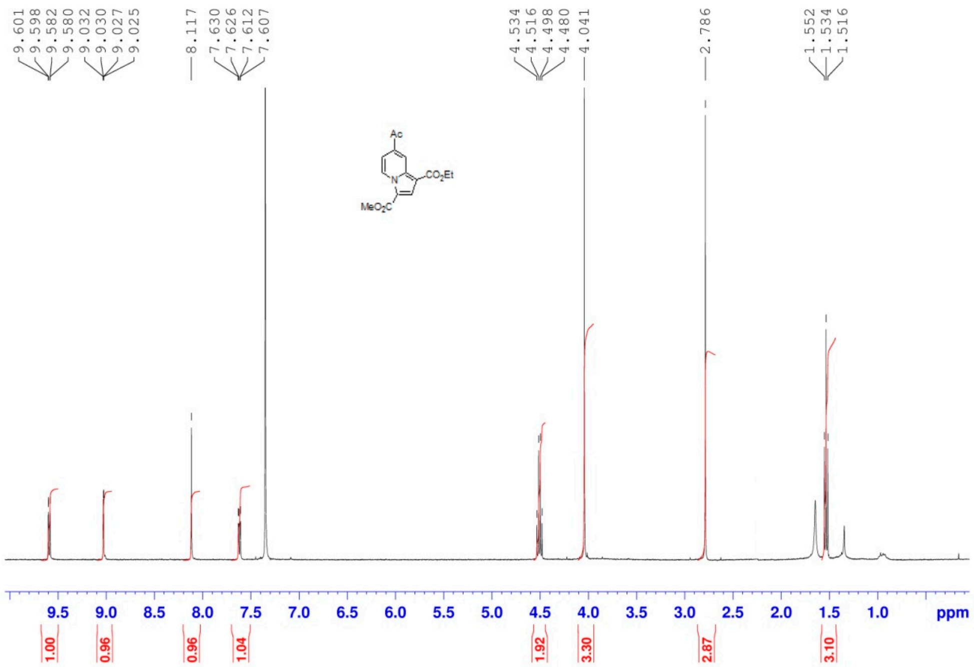

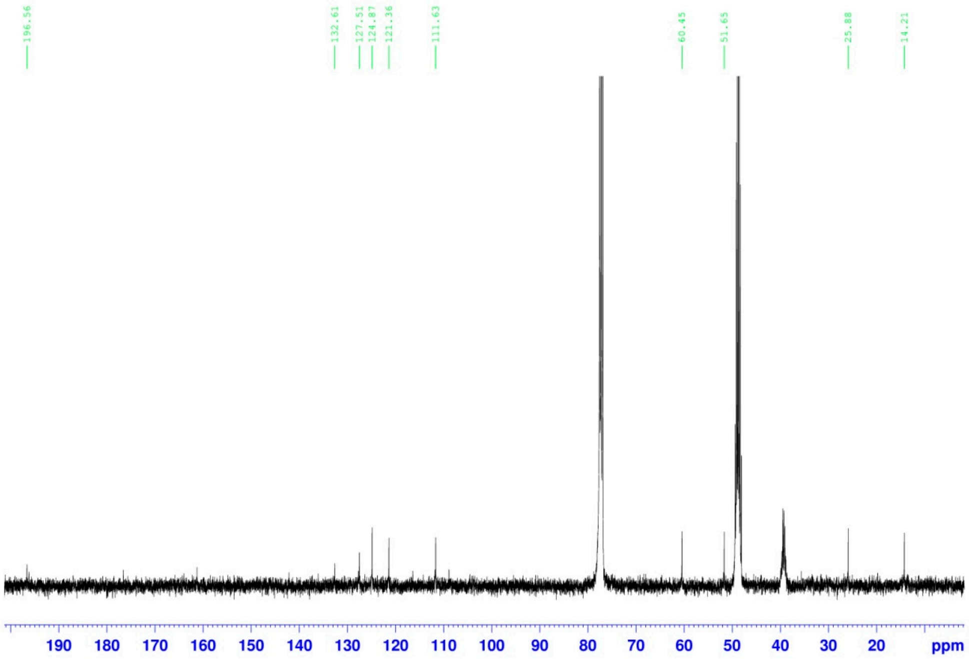
196.56
132.61
127.51
124.87
121.36
111.63
60.45
51.65
25.88
14.21
190
180
170
160
150
140
130
120
110
100
90
80
70
60
50
40
30
20
ppm

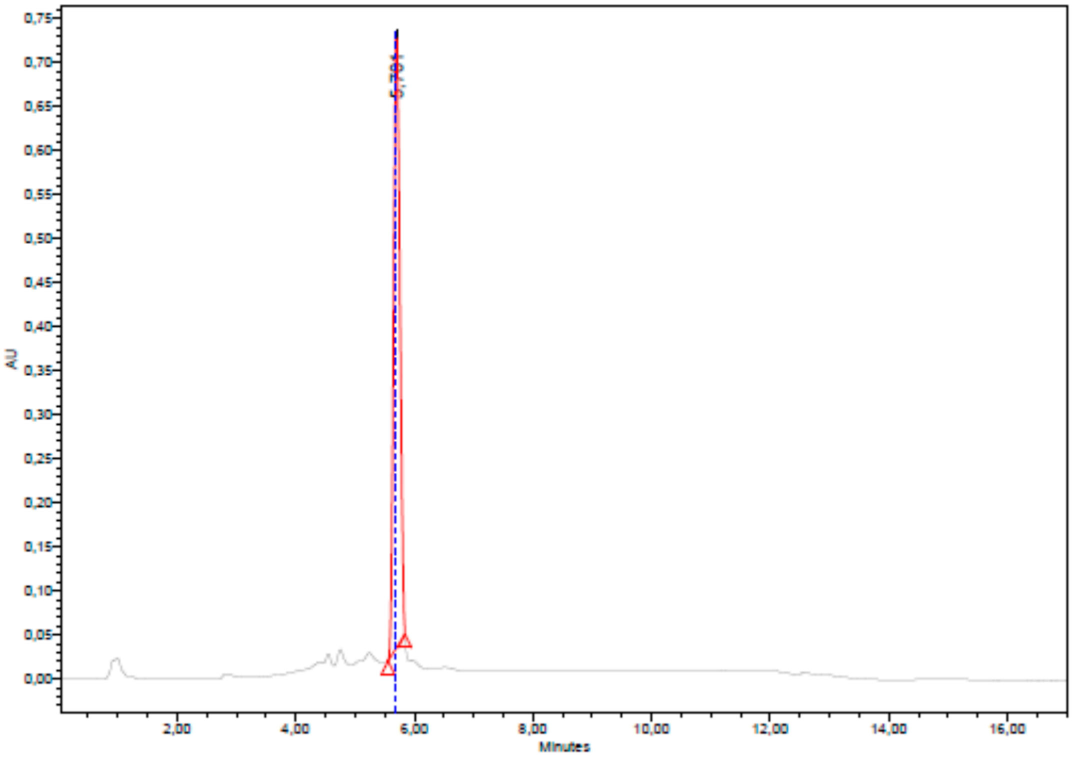
AU
Minutes
5,701
0,00
0,05
0,10
0,15
0,20
0,25
0,30
0,35
0,40
0,45
0,50
0,55
0,60
0,65
0,70
0,75
2,00
4,00
6,00
8,00
10,00
12,00
14,00
16,00

1-Ethyl 3-methyl 7-(trifluoromethyl)indolizine-1,3-dicarboxylate (19a) and 1-methyl 3-methyl 7-(trifluoromethyl)indolizine-1,3-dicarboxylate (19b) (30/70 ratio)

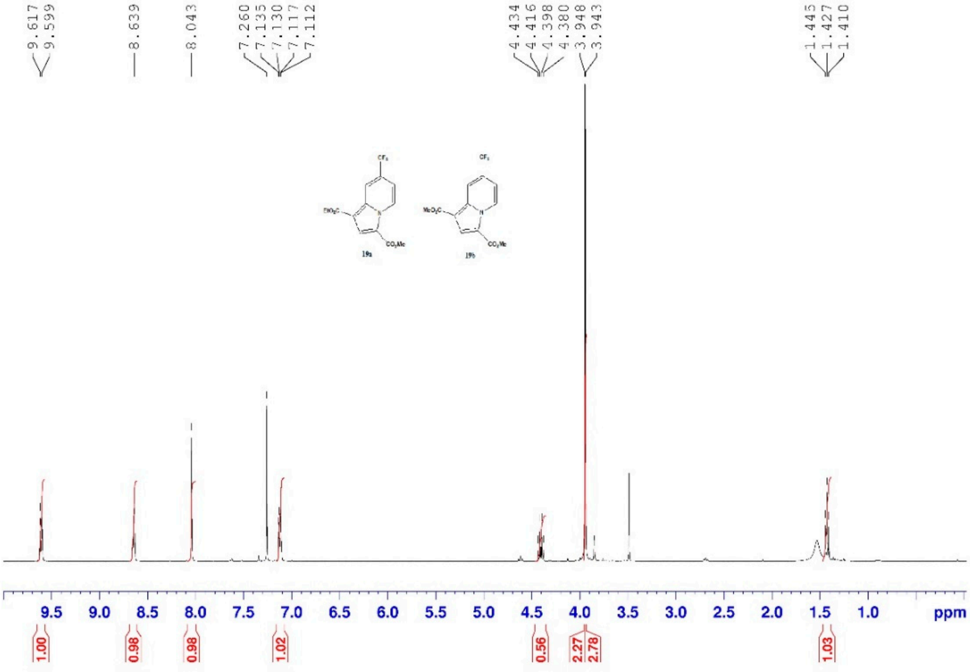

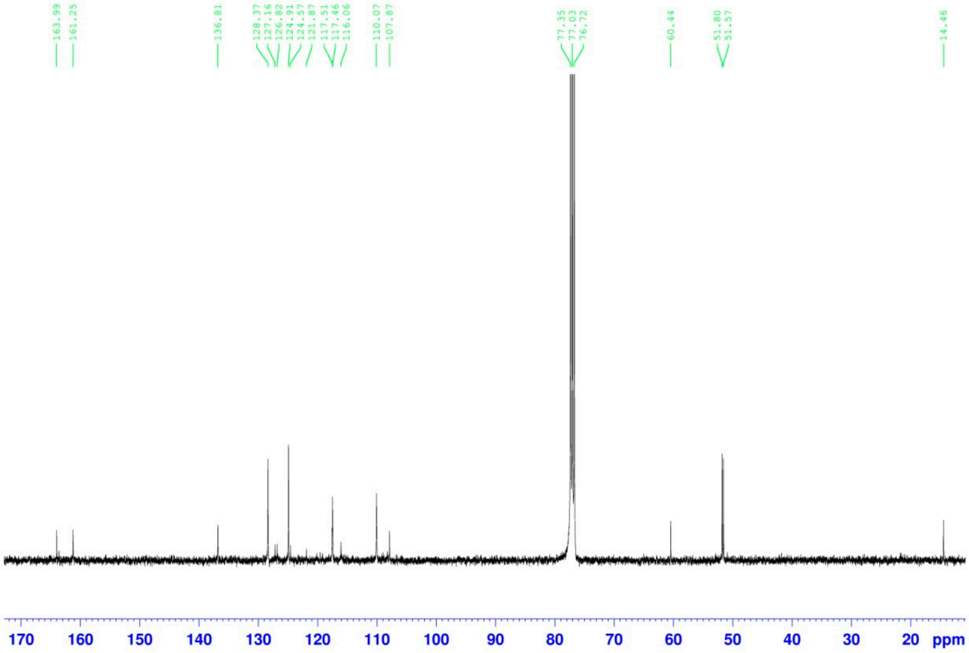

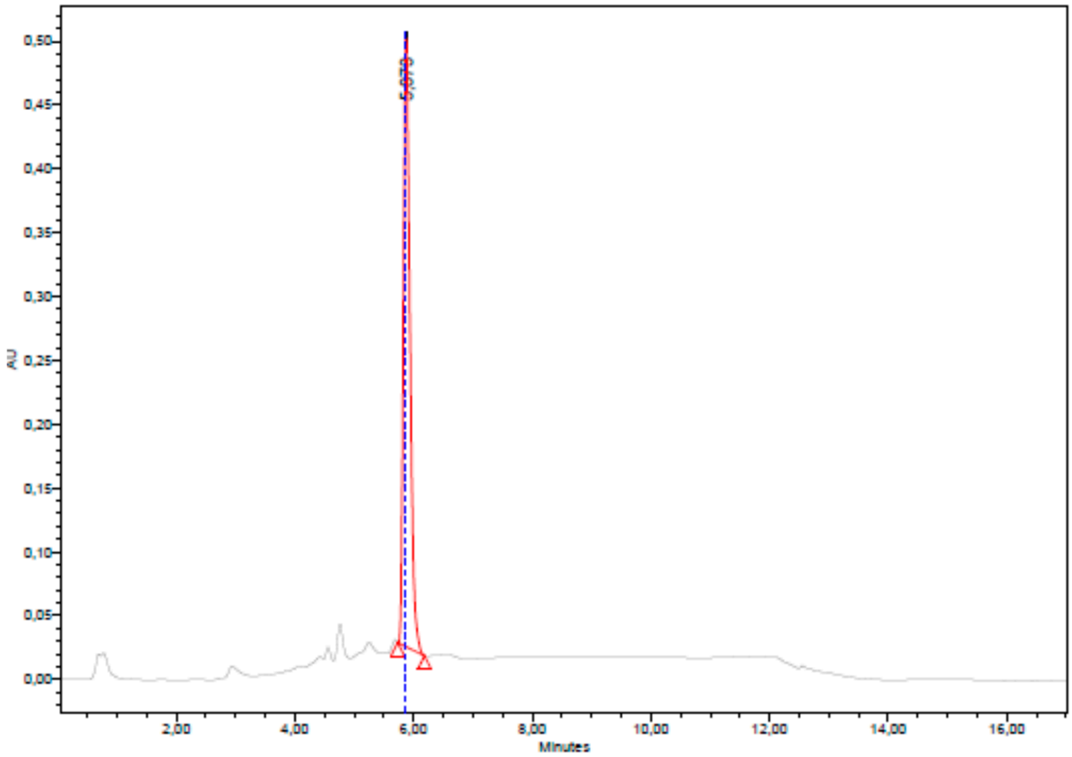

Ethyl 3-methyl 7-cyanoindolizine-1,3-dicarboxylate (20)

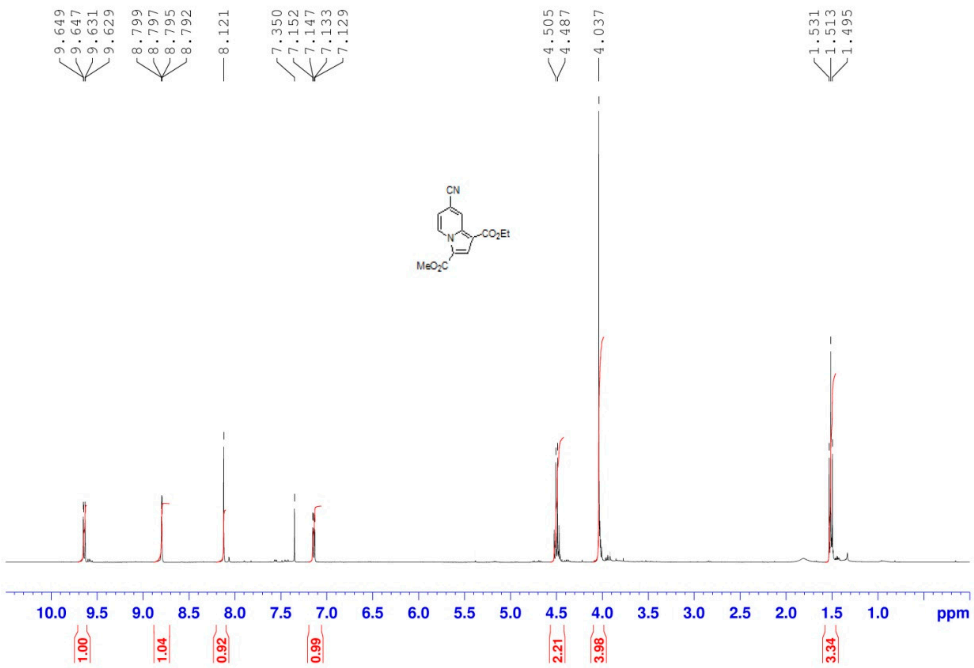

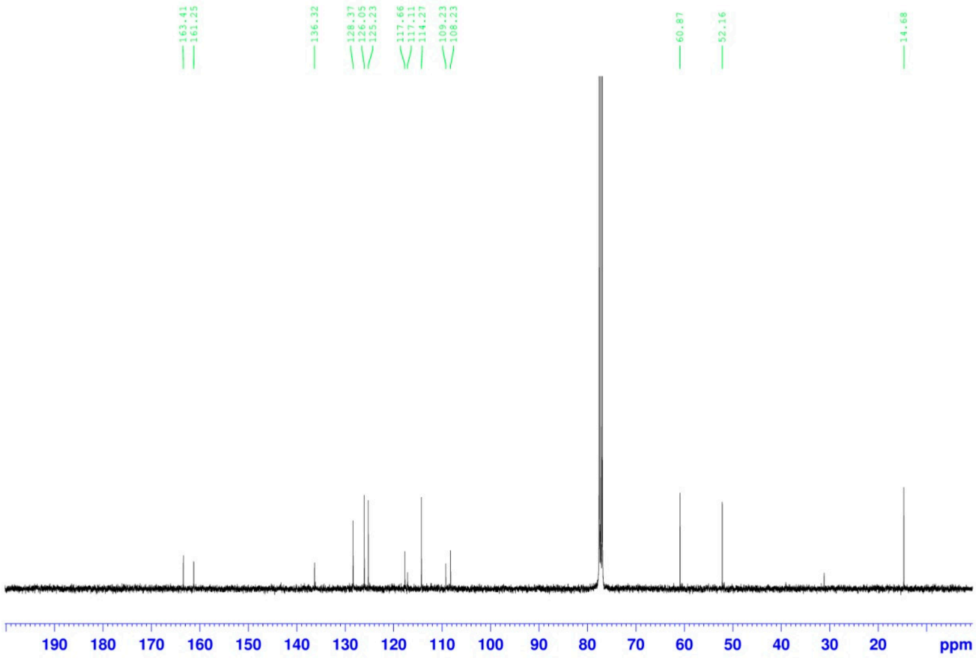
163.41
161.25
136.32
128.37
126.05
125.23
117.66
117.11
114.27
109.23
108.23
60.87
52.16
14.68
190 180 170 160 150 140 130 120 110 100 90 80 70 60 50 40 30 20 ppm

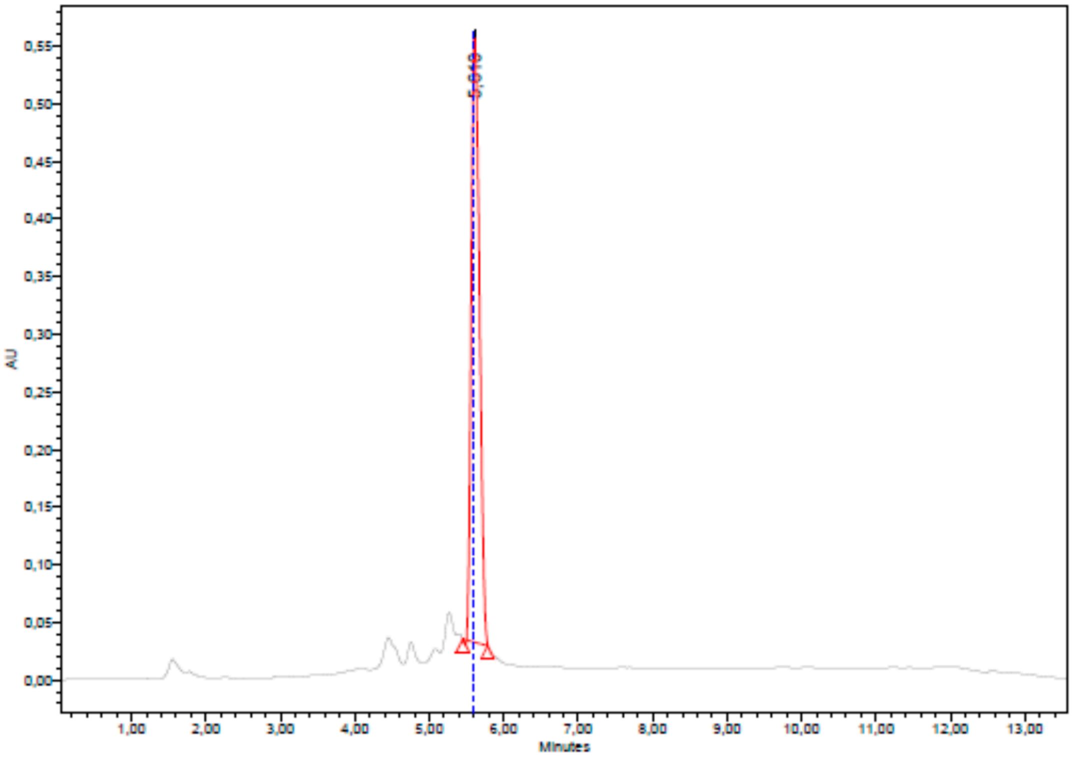
AU
Minutes
0,55
0,50
0,45
0,40
0,35
0,30
0,25
0,20
0,15
0,10
0,05
0,00
1,00 2,00 3,00 4,00 5,00 6,00 7,00 8,00 9,00 10,00 11,00 12,00 13,00

1,3-Diethyl 7-acetylindolizine-1,3-dicarboxylate (22)

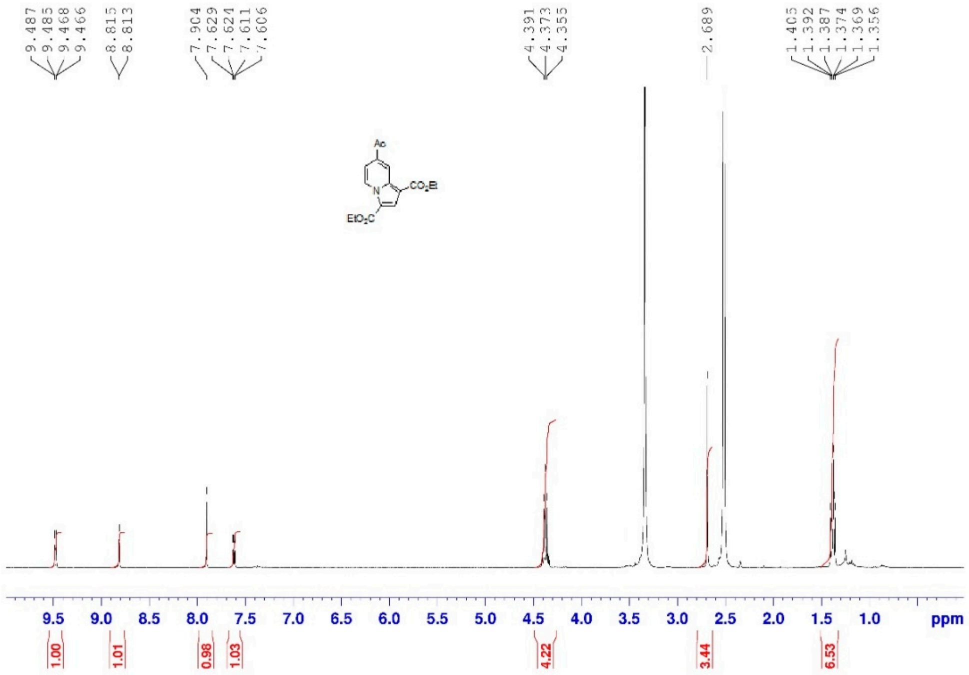

1,3-Diethyl 7-cyanoindolizine-1,3-dicarboxylate (23)

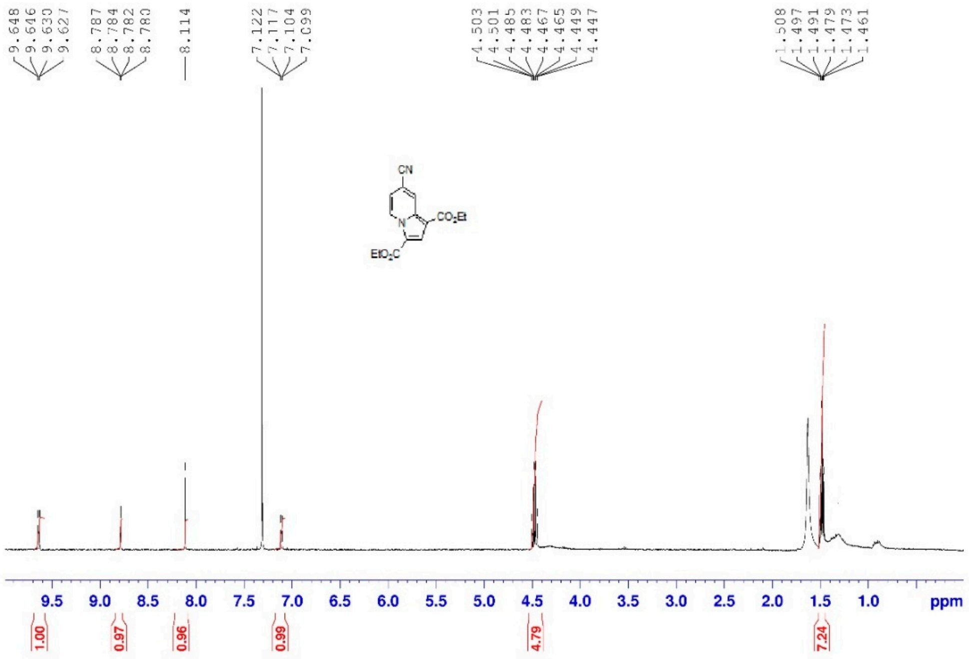

1-Ethyl 3-benzoyl-7-cyanoindolizine-1-carboxylate (24)

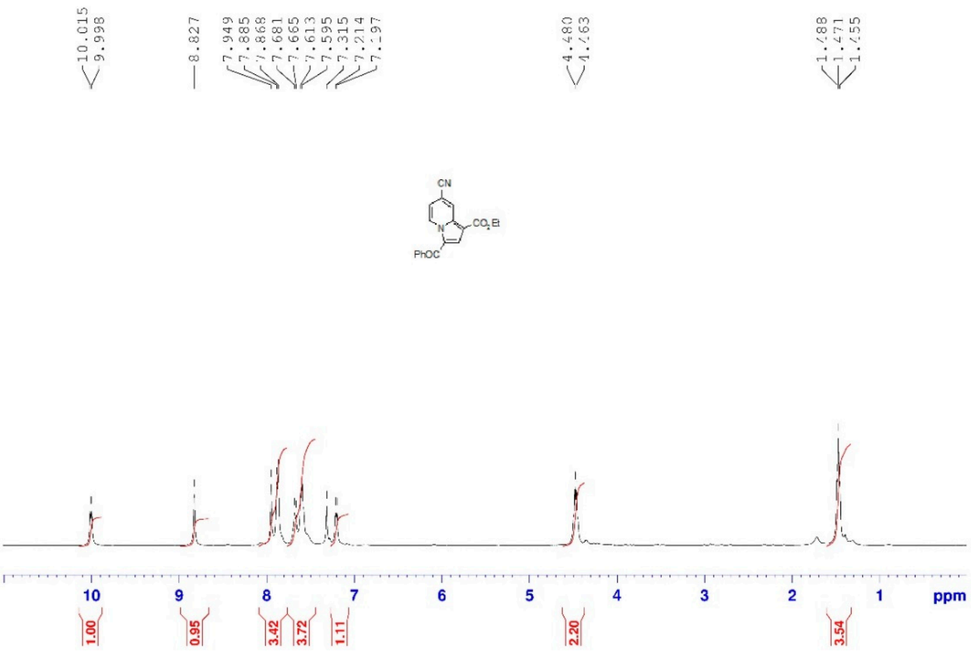

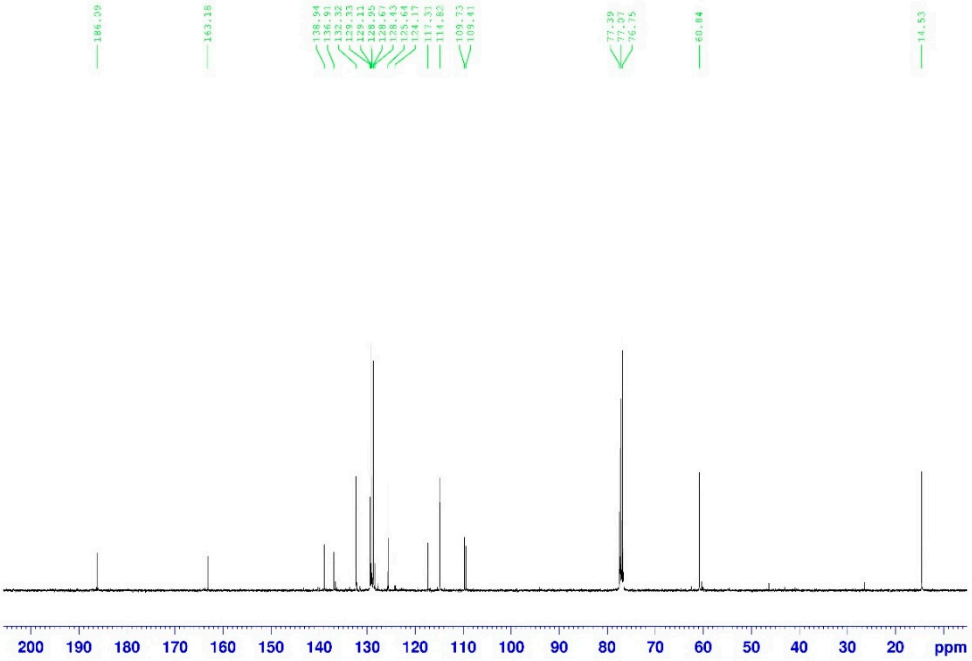

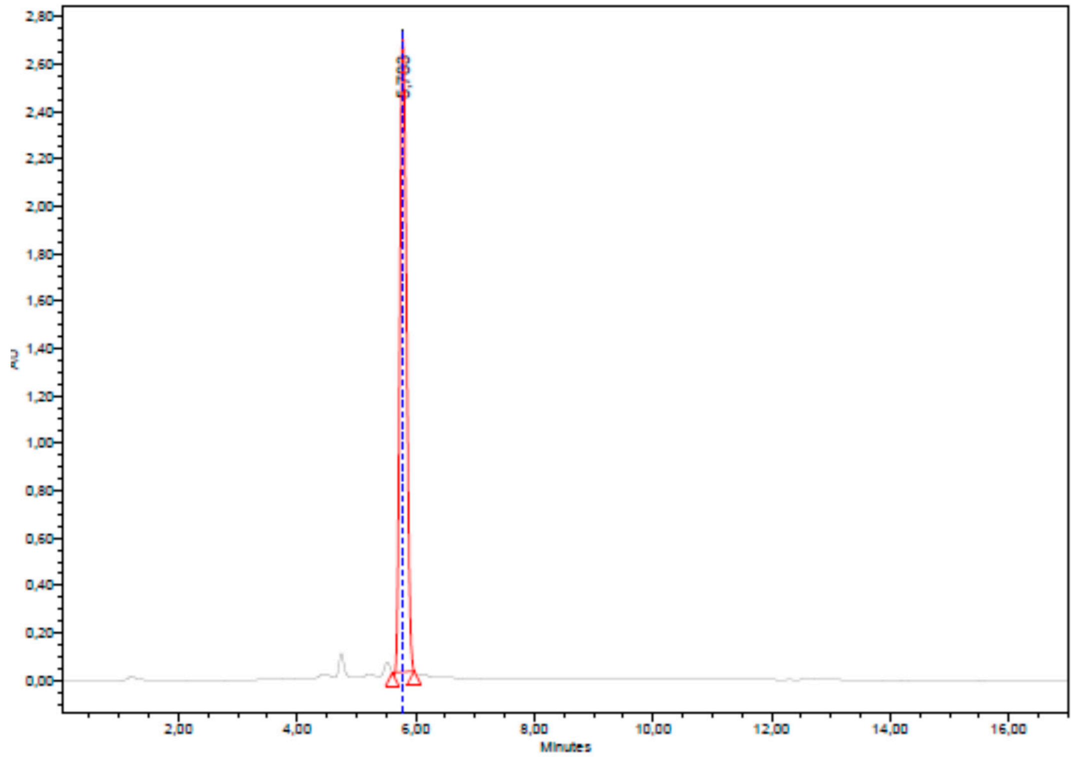

1-Ethyl 3-(4-nitrobenzoyl)-7-cyanoindolizine-1-carboxylate (25)

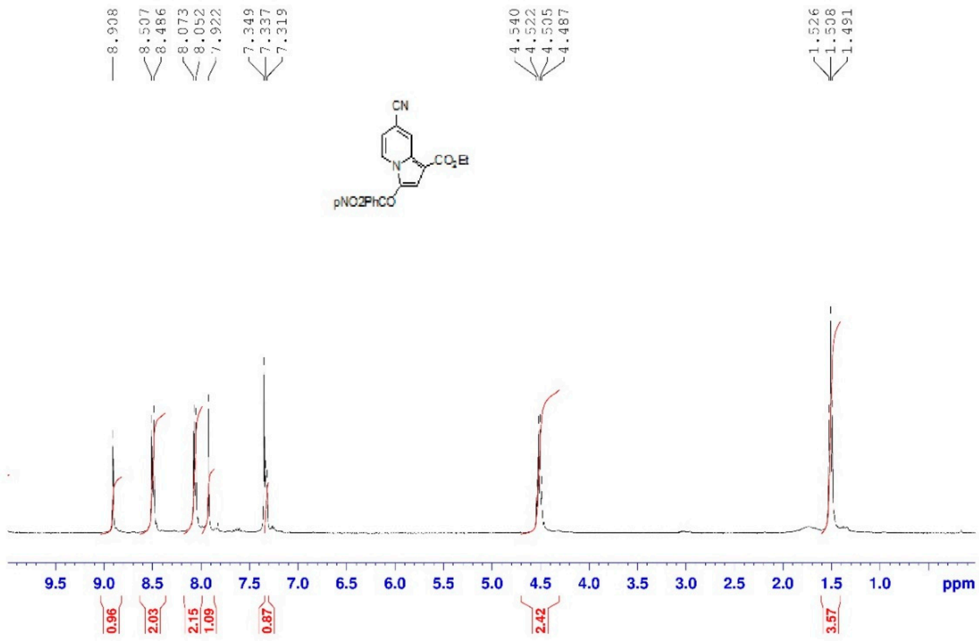

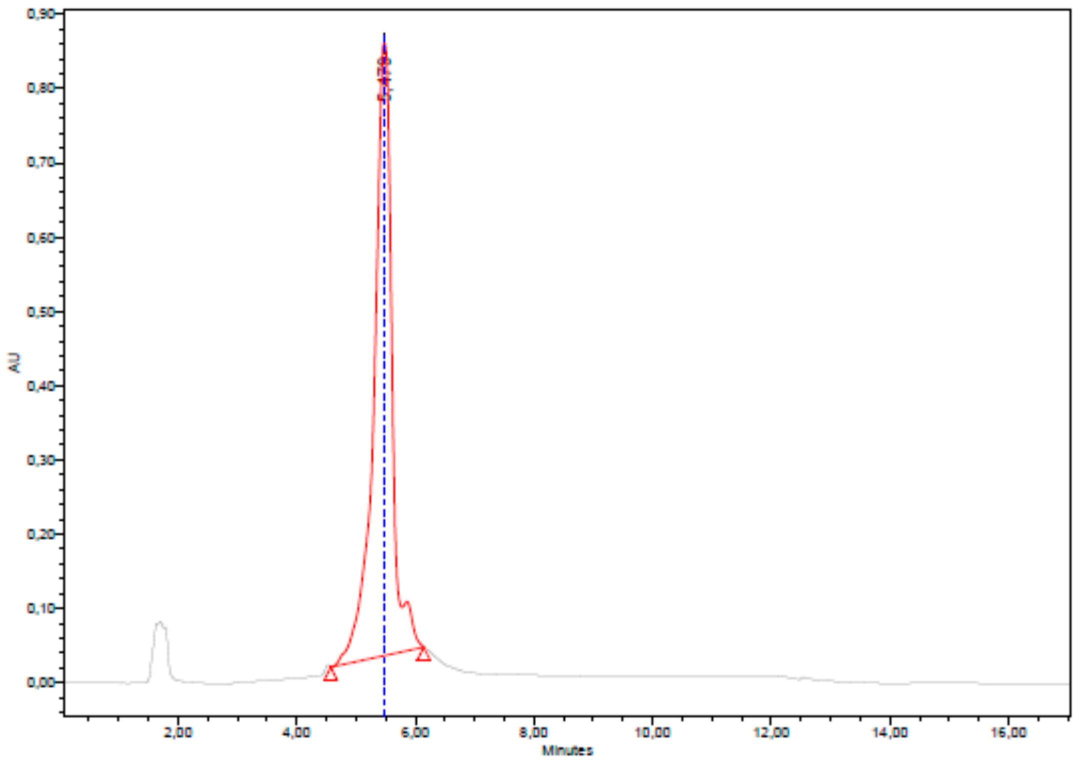

1-Ethyl 7-cyano-3-(*N*-propylcarbamoyl)indolizine-1-carboxylate (26)

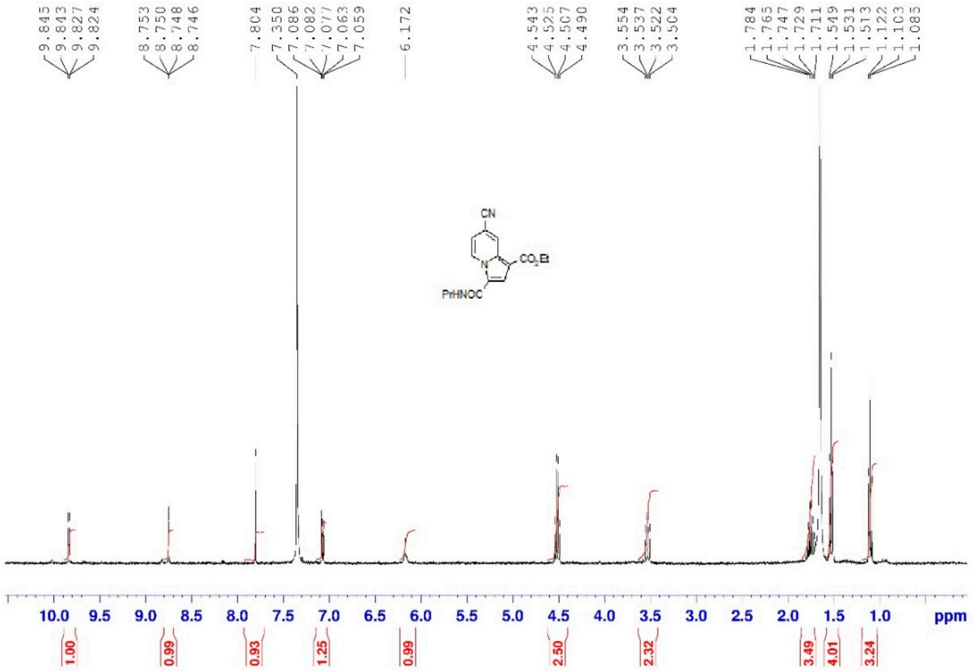

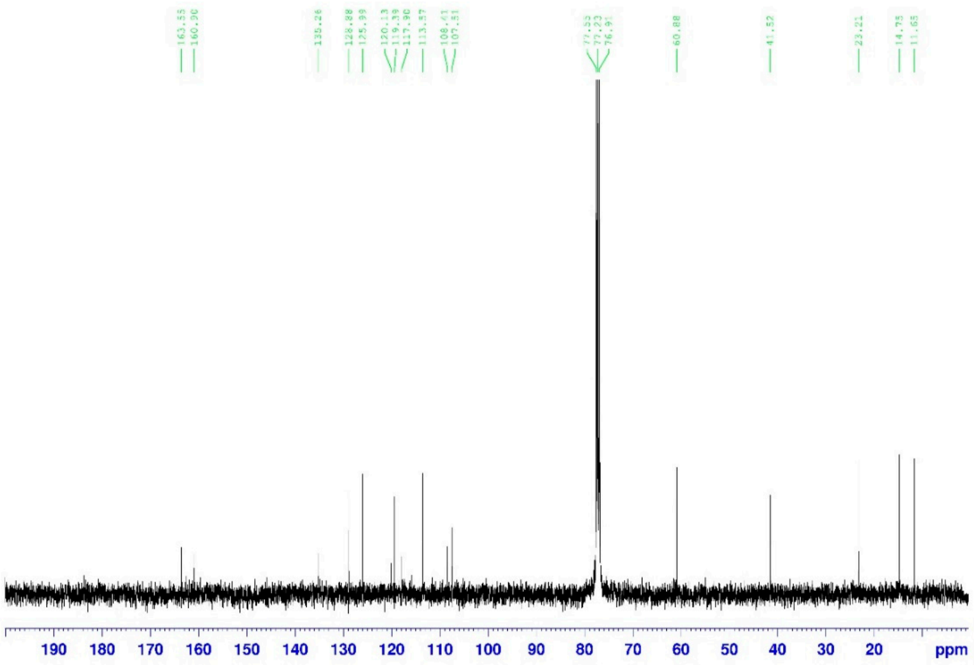
163.55
160.90
135.26
128.88
125.99
120.13
119.39
117.90
113.57
108.41
107.51
77.55
77.23
76.91
60.88
41.52
23.21
14.75
11.65
190 180 170 160 150 140 130 120 110 100 90 80 70 60 50 40 30 20 ppm

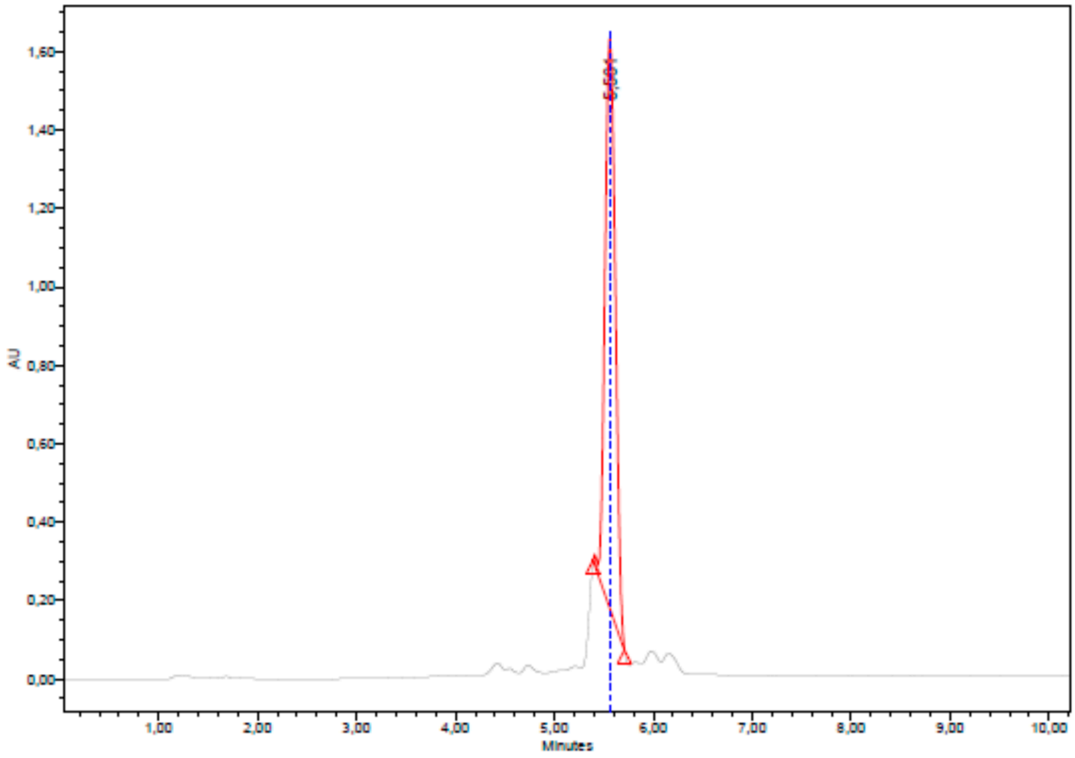
AU
1,60
1,40
1,20
1,00
0,80
0,60
0,40
0,20
0,00
1,00 2,00 3,00 4,00 5,00 6,00 7,00 8,00 9,00 10,00
Minutes

Methyl 1-(*N*-benzylcarbamoyl)-7-cyanoindolizine-3-carboxylate (28)

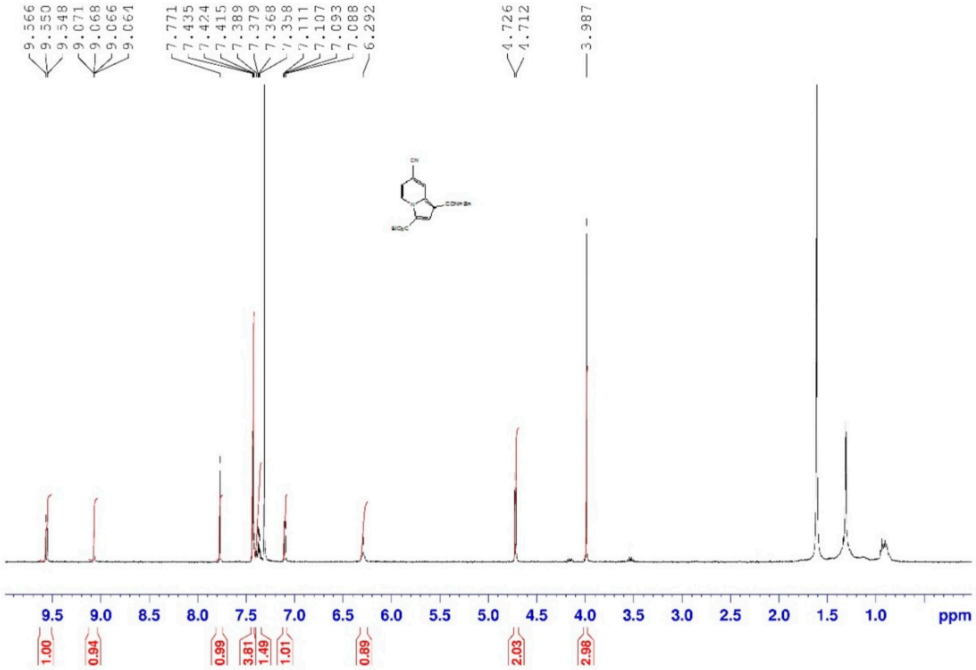

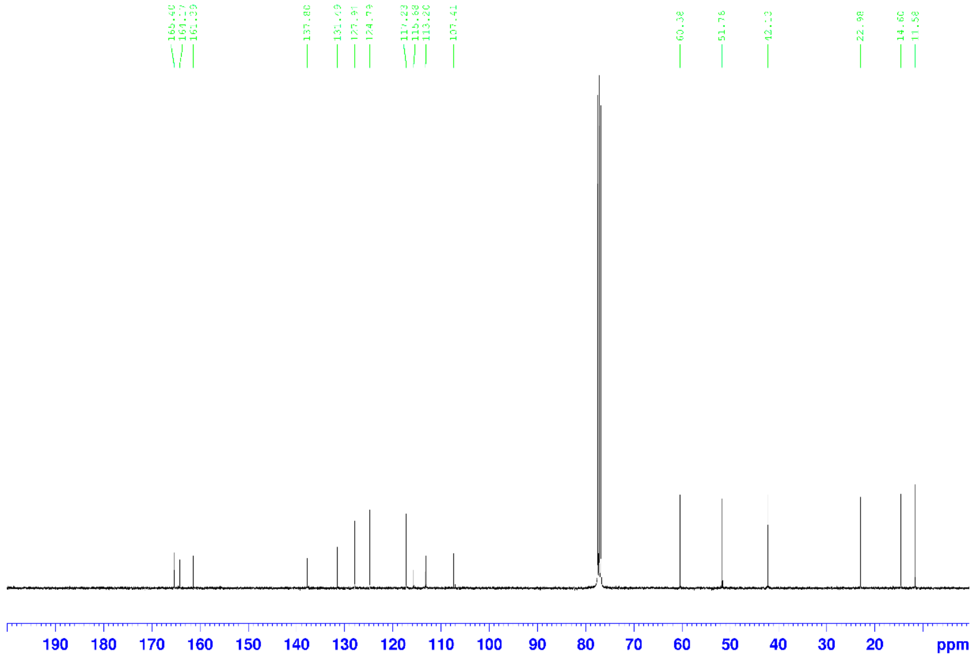

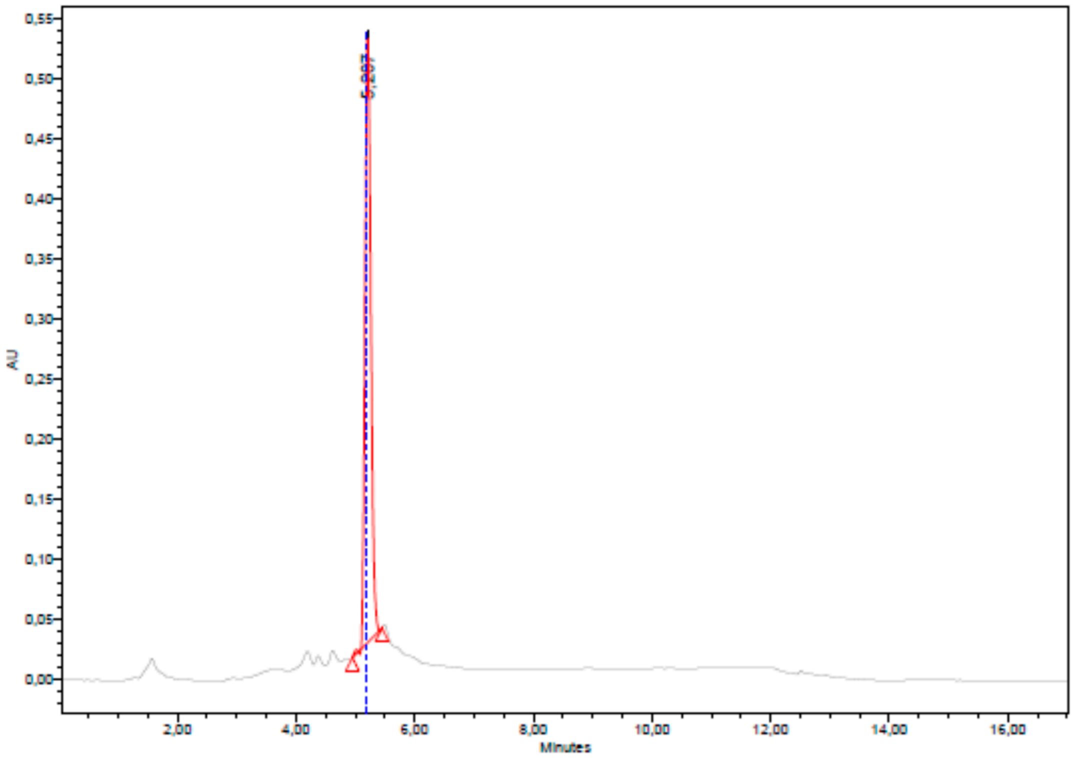

AU
Minutes
5,207
